# Functionalized metal-organic framework and MOF-derived materials for bone regeneration applications

**DOI:** 10.3389/fbioe.2025.1645657

**Published:** 2025-08-29

**Authors:** Yuesen Fan, Chengbin Long, Yuyi Cai, Yingkun Hu, Lihua Peng

**Affiliations:** ^1^ Chongqing Medical University, Chongqing, China; ^2^ Department of Orthopedics, Bishan Hospital of Chongqing Medical University, Chongqing, China; ^3^ Department of Orthopedics, Bishan Hospital of Chongqing, Chongqing, China; ^4^ Department of Stomatology, Daping Hospital, Army Medical University (The Third Military Medical University), Chongqing, China; ^5^ Department of Orthopedics, The First Affiliated Hospital of Chongqing Medical University, Chongqing, China; ^6^ Orthopedic Laboratory, Chongqing Medical University, Chongqing, China

**Keywords:** metal-organic framework (MOF), nanomaterials, bone regeneration, bone repair, biomaterials

## Abstract

Bone defects resulting from trauma, tumors, infections, and aging present significant clinical challenges, with conventional grafts hindered by limitations in biocompatibility, mechanical strength, and integration. Metal-organic frameworks (MOFs), as advanced nanomaterials with tunable porosity, high surface area, and stimuli-responsive properties, hold immense potential for bone regeneration. This review provides a comprehensive overview of the classification, synthesis methods, osteogenic mechanisms, and applications of functionalized MOFs and their derivatives in bone repair. MOFs are classified based on structural topology, chemical composition, and functional applications. Synthesis techniques, including solvothermal, ultrasonic, and electrochemical approaches, are evaluated for customizing physical properties such as pore architecture and stability. Osteogenic mechanisms encompass enhancing implant physical characteristics to promote cell adhesion, sustained release of metal ions to activate signaling pathways, controlled drug delivery for targeted therapy, and anti-inflammatory/antioxidant effects through reactive oxygen species scavenging. Applications address various bone pathologies, demonstrating improved angiogenesis, osteointegration, and antibacterial performance in preclinical studies. Key challenges, including cytotoxicity, long-term biosafety, and scalability, are discussed, alongside strategies like surface modification and hybrid composites to overcome these barriers. Future perspectives focus on developing smart MOF-based scaffolds for personalized regenerative medicine, underscoring their transformative potential in orthopedic therapies.

## 1 Introduction

The regenerative capacity of bone tissue originates from the special structure of the Haversian system. Its regeneration process involves the precise synergy between osteoblasts and osteoclasts. This dynamic remodeling mechanism is precisely the research focus of orthopedic regenerative medicine. With the increasingly evident trend of population aging, the restoration of bone damage due to factors such as trauma, tumors, and infections has become an important challenge in clinical practice (Duda et al., 2023). Globally, there are approximately 178 million new fractures each year, with around 5% of these developing fracture-related infections (FRIs), corresponding to roughly 1.8 million FRI cases annually (Wu A.-M. et al., 2021). The hospitalization costs for patients with FRIs are 4–8 times higher than those for patients without infections (Metsemakers et al., 2024). Notably, low- and middle-income countries (LMICs) and conflict regions bear a heavier economic burden associated with infections, primarily due to the higher proportion of open fractures in these areas (Tissingh et al., 2022). In modern orthopedic clinical practice, a diversified treatment strategy system has been formed for complex bone defect cases, including but not limited to treatment modalities comprising self-donated bone grafts, donor bone grafts, and bone graft replacements, and a single or combined intervention plan can be implemented according to individual pathological characteristics (Qing et al., 2020; Zhao D. et al., 2021; Liu X. et al., 2022). Although the existing clinical strategies have made phased progress, the traditional bone graft substitutes still face bottleneck problems such as biocompatibility limitations, insufficient mechanical strength, and low bone integration efficiency (Li Z. et al., 2023). These bottlenecks prompt researchers to turn their attention to the field of nanotechnology.

Currently, clinical practice employs a range of therapeutic approaches for functional restoration and bone defect healing, including metal implants, autografts, and allografts—strategies widely acknowledged as the “gold standard” in this field. Additionally, interventions such as vascularized bone grafting, autologous chondrocyte transplantation, and joint replacement are frequently utilized to address clinical needs related to bone defects. However, these conventional methods are plagued by inherent limitations. For metal implants, the necessity of surgical removal in certain cases may pose a risk of secondary trauma to patients. Autografts, while effective, are hampered by limited donor supply, complications at the donor site, and heightened risks of surgical site infections, all of which can induce significant patient discomfort. Similarly, allografts are associated with potential hazards such as disease transmission and challenges of immune rejection, which compromise their clinical utility. Consequently, there is an urgent imperative to develop reliable novel therapeutic modalities that can address the unmet medical demands in bone tissue defect repair, while alleviating the discomfort and adverse effects associated with current bone regeneration strategies (Xie et al., 2021; Maia et al., 2022; Ramanathan et al., 2024; Wang et al., 2025b).

Nanomaterials have advantages such as multi-scale structure regulation, functional synergy, and intelligent response characteristics. When combined with artificial bone scaffolds, they can make up for the current deficiencies of artificial bone scaffolds (Feng et al., 2023). Nanomaterials are systematically classified into distinct dimensional categories: 0D (molecular), 1D (chain-like), 2D (layered), and 3D (network). Materials like quantum dots and gold nanoparticles, which have dimensions between 1 and 100 nm, are known as 0D nanomaterials. 1D materials refer to those with one dimension beyond the nanoscale, such as silicon nanowires and carbon nanotubes. 2D materials refer to those with two dimensions beyond the nanoscale, such as graphene and molybdenum disulfide nanosheets. 3D materials are bulk materials composed of nanostructured units, such as metal-organic frameworks (MOFs) and nanocomposites. Nanomaterials provide significant advantages in bone tissue engineering, thanks to their extensive specific surface area, active nature, and capacity to precisely modulate their physical and chemical characteristics. In recent years, the critical research frontier has been the application of functional nanomaterials in therapies aimed at improving bone regeneration and repair (Zhu et al., 2020; Babuska et al., 2022). MOFs represent a category of nanomaterials characterized by coordination compounds that arise from the interaction between organic ligands and metal ions or clusters. While retaining the inherent advantages of nanomaterials, their porous architecture allows for the diffusion of guest molecules that respond to stimuli, giving them dynamic responsiveness to external triggers, which is highly useful for various biomedical applications (Guan et al., 2023). Due to their customizable structures and adjustable sizes, MOFs and their composite materials can achieve targeted delivery and controlled release after being designed and characterized. Compared to standalone ionic therapies, this approach enhances ion utilization efficiency while mitigating side effects caused by fluid diffusion (Yang Q. et al., 2024).

MOFs constitute an emerging class of hybrid porous materials, characterized by infinite crystalline lattices assembled via coordinate bond interactions between organic ligands—functioning as bridging linkers—and metal ions that serve as nodal centers in the structural framework (Binaeian et al., 2023; Sezgin et al., 2025). MOFs have demonstrated applicability across diverse fields, including chemical engineering, materials science, energy storage, sensing, pollution remediation, and biomedical applications (Yang and Yang, 2020; Zhou et al., 2021; Lin R. et al., 2023; Guo et al., 2024; Molavi et al., 2024). To date, a variety of MOFs have been designed based on their component units, among which the most widely used are Porous Coordination Networks (PCNs), Materials Institute Lavoisier (MIL) MOFs, Zeolitic Imidazolate Frameworks (ZIFs), Isoreticular MOFs (IRMOFs), University of Oslo (UIO) MOFs, and Porous Coordination Polymers (PCPs) (Table 1) (Yusuf et al., 2022; Zhang X. et al., 2022). PCNs are stereo-octahedron materials, consisting of multiple cuboctahedral nanocages that form a cage-pore channel architecture in three-dimensional space, making it suitable for gas storage (Hou et al., 2021). MIL MOFs were initially synthesized using organophosphates or succinic acid to link central metal ions, but modern synthesis primarily utilizes carboxylates and triply charged metal ions comprising iron(III), aluminum(III), gallium(III), indium(III), vanadium(III), and chromium(III) (Zhang H. et al., 2022). The synthesis of ZIFs involves reacting Zn^2+^ or Co^2+^ with imidazole ligands, producing porous crystalline materials that have zeolite-like tetrahedral frameworks (Liu et al., 2022c). IRMOFs are assembled from [Zn_4_O]^6^+ clusters and aromatic carboxylate ligands, resulting in octahedral microporous crystals possessing three-dimensional porous frameworks that allow for functionalization with organic groups (Wu et al., 2024). UIO MOFs are three-dimensional microporous materials formed by the coordination of [Zr_6_O_4_(OH)_4_] clusters with BDC ligands, featuring an octahedral central pore cage surrounded by eight tetrahedral corner cages (Huo et al., 2023). Porous coordination polymers (PCPs) are structured from transition metal ions, in which carboxylic acids, pyridines, and their derivatives play dual roles as both primary building units (PBUs) and secondary building units (SBUs) in the formation of their framework architecture (Tong et al., 2020). Furthermore, based on the aforementioned classification, Some MOF materials derive their names from the universities affiliated with their researchers. These materials generally possess distinct structures and properties, and have typically undergone extensive research and found widespread application, such as, Dresden University of Technology (DUT-n) (Mendt et al., 2022), Nanyang University of Technology (NTU-n) (Crespí Sánchez et al., 2021), Hong Kong University of Science and Technology (HKUST-n) (Jagódka et al., 2022), Beijing Jianzhu University (BUC-n) (Zhao et al., 2020), Pohang University of Science and Technology (POST-*n*) (Harambage, 2025), Christian-Albrechts-University (CAU-n) (Giri et al., 2025), Northwestern University (NU) (Abazari et al., 2024). Simultaneously, Concurrently, a novel category of metal-organic frameworks has emerged, which integrates the fundamental principles of MOFs with biological sciences, thus coining the novel designation “Bio-MOFs”. These Bio-MOFs present compelling opportunities and promising prospects across interdisciplinary research domains (McKinlay et al., 2010). Certain researchers have established specific criteria for classifying metal-organic frameworks as Bio-MOFs, with a core criterion being the incorporation of at least one biomolecular unit that functions as an organic ligand in their framework (Cai et al., 2019). The significant development has also been achieved in therapeutic agents formed by combining active ligands—such as amino acids (Lyu et al., 2022), peptides (Wang S. et al., 2022), proteins (Sontz et al., 2015), nucleobases (Chand et al., 2022), saccharides (carbohydrates) (Di Palma et al., 2022), drugs (Li W. et al., 2024), and other bioactive molecules (Chen J. et al., 2021)—with active metals. Recently, these MOF architectures have been extensively investigated as promising platforms for biomedical applications (Ma et al., 2023). Owing to their outstanding chemical and physical traits, MOFs have emerged as a focal point for extensive research with their application scope including biosensing platforms, efficient drug delivery materials (Horcajada et al., 2006), the design of quantum devices (Gimeno et al., 2025), and utilization as catalysts (Pascanu et al., 2019). Moreover, many new MOF variants currently being developed have attracted considerable interest lately, with their uses increasingly spreading across various fields (Coluccia et al., 2022; Cun et al., 2022).

**TABLE 1:** Types of MOF terms.

Terms	Abbreviation	Ref
UiO-66	University of Oslo	Yang et al. (2011)
IRMOF-1	Isoreticular Metal–Organic Frameworks	Gatou et al. (2023)
IRMOF-3		Wu et al. (2024)
Zr-MOF		Yan et al. (2022)
NU-1012	Northwestern University	Wang et al. (2022d)
MOF-5	Metal–Organic Frameworks	Eddaoudi et al. (2002)
Eu-MOF		Xu et al. (2022)
DUT-4	Dresden University of Technology	Stillman et al. (2023)
DUT-5		Stillman et al. (2023)
MIL-101	Materials Institute Lavoisier	Zhang et al. (2018a)
MIL-100		Lin et al. (2023c)
MIL-53		Yuan et al. (2023b)
MIL-88		Arenas-Vivo et al. (2020)
Eu-BTC MOF		Lo Presti et al. (2023)
Fe-MOFs		Zheng et al. (2023)
ZIF-67	Zeolite Imidazolate Framework	Wu et al. (2022b)
ZIF-90		Hsu et al. (2021)
ZIF-8		Ho et al. (2020)
ZIF-78		Banerjee et al. (2009)
ZIF-100		Wang et al. (2008)
Ce-SINAP-1		Wu et al. (2021b)
PCN-14	Porous Coordination Networks	Ma et al. (2008)
PCN-222		Chen et al. (2021b)
Co-PMOF		Zhang et al. (2019)
Y-MOF		Wang et al. (2023)
CD-MOF	Cyclodextrin Metal–Organic Frameworks	Hamedi et al. (2022)
spe-MOF		Rao et al. (2025)
MOF 1		Li et al. (2021a)
RhCu-rht-MOF		Grancha et al. (2021)
HKUST-1	Hong Kong University of Science and Technology	Zhao et al. (2024a)
NTU-9	Nanyang University of Technology	Crespí Sánchez et al. (2021)
BUC-21	Beijing Jianzhu University	Zhao et al. (2020)

**FIGURE 1:**
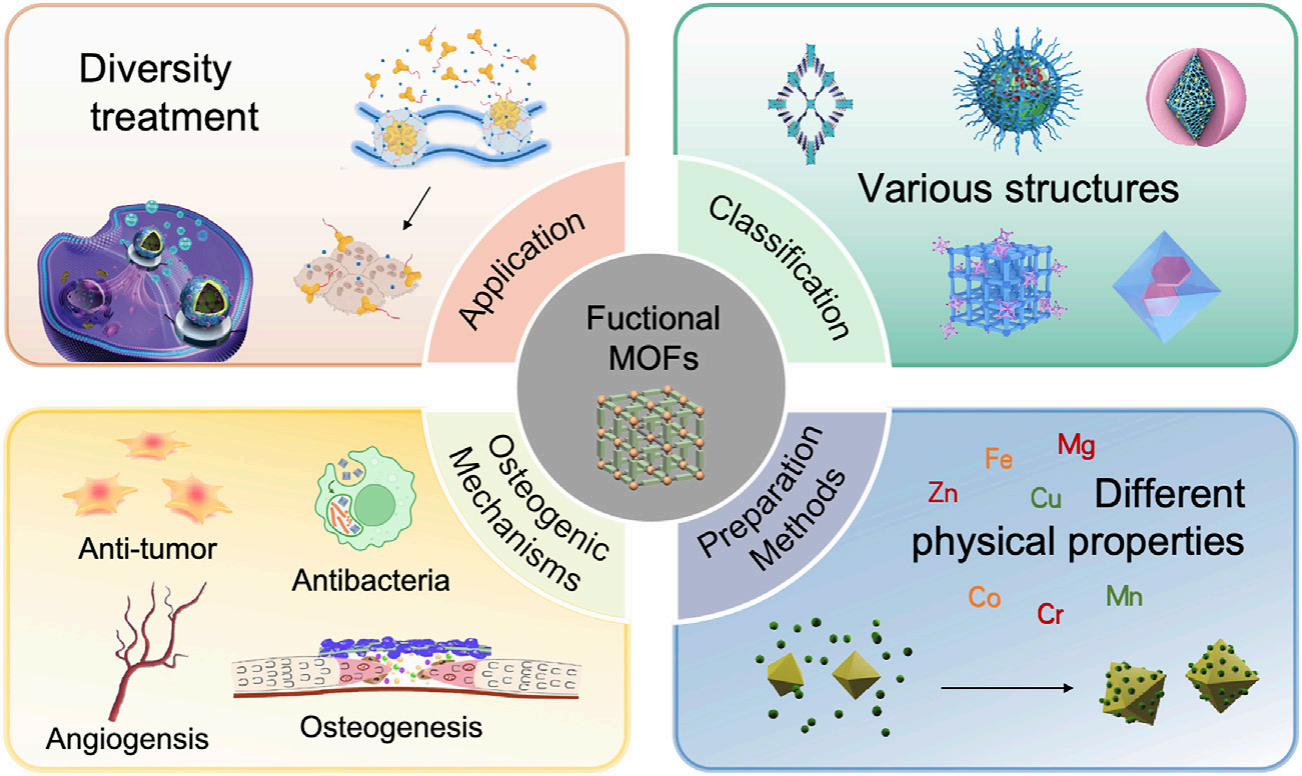
The schematic overview of the classification, synthesis, and applications of MOFs in the regulation of osteogenesis. The figure was created using Biorender.

MOFs have emerged as prominent materials in the biomedical sector, particularly for applications that promote bone growth (Zheng et al., 2024), due to their exceptional functionalities and customizable characteristics, such as the Precise regulation of drug loading capacity and release kinetic profiles (Zhang et al., 2024). This capability for controlled delivery facilitates sustained or stimuli-responsive release from nanoparticle-based drug formulations or functional coatings (Wang Y. et al., 2020), Meanwhile, through more efficient bone-targeting capabilities and the delivery of multiple osteogenesis-promoting pharmaceutical components (Salcedo-Abraira et al., 2025), it addresses the limitations of drug therapies for diseases such as osteoporosis and bone metastasis, which are constrained by insufficient bone tissue perfusion and inadequate drug concentration at lesion sites (Vassaki et al., 2021; Pan et al., 2022). Additionally, it mitigates the various side effects caused by increased therapeutic doses, including hypocalcemia, osteonecrosis of the jaw, bone pain, and osteomyelitis (Foessl et al., 2023), thereby significantly enhancing their therapeutic precision in bone regeneration (Figure 1). Numerous studies and reviews within this field have investigated the composition, structure, and mechanisms of bone growth associated with various MOFs, demonstrating that MOFs and their derivatives exhibit significant osteogenic activity. The successful incorporation of these materials into bone implants is critically contingent upon the optimization of dosage and structural configurations, as substantiated by empirical evidence in the literature (Zulfiqar et al., 2022; Zhang et al., 2023; Ghovvati et al., 2024). Nevertheless, the potential cytotoxicity of this compound has constrained its clinical application, posing a significant challenge in concurrently augmenting its osteogenic activity and biocompatibility. This review provides a comprehensive summary of the classification, synthesis methods, and mechanisms by which MOFs enhance bone healing. Recent advancements in MOF research have elucidated that their osteogenic mechanisms are predominantly attributed to the following factors: (1) physical interactions, such as nanotopography-guided cell adhesion; (2) the release of ionic components, specifically Ca^2+^ and Zn^2+^, which mediate signaling pathways; (3) the delivery of drug payloads, including growth factors and siRNA; and (4) their anti-inflammatory and antioxidant properties, particularly through reactive oxygen species (ROS) scavenging. This paper examines the applications of MOFs in various bone pathologies and addresses the critical challenges associated with their use, particularly focusing on long-term biosafety and degradation kinetics. We review strategies to optimize the healing performance of MOFs from biomechanical, topological, and physicochemical perspectives. This includes approaches to enhance biocompatibility, refine synthesis protocols, modulate surface roughness and pore architecture, and engineer MOF-based composites. Finally, we offer forward-looking perspectives on emerging trends, emphasizing the potential of MOF-based materials in advanced bone healing applications.

## 2 MOFs and their classification

**FIGURE 2:**
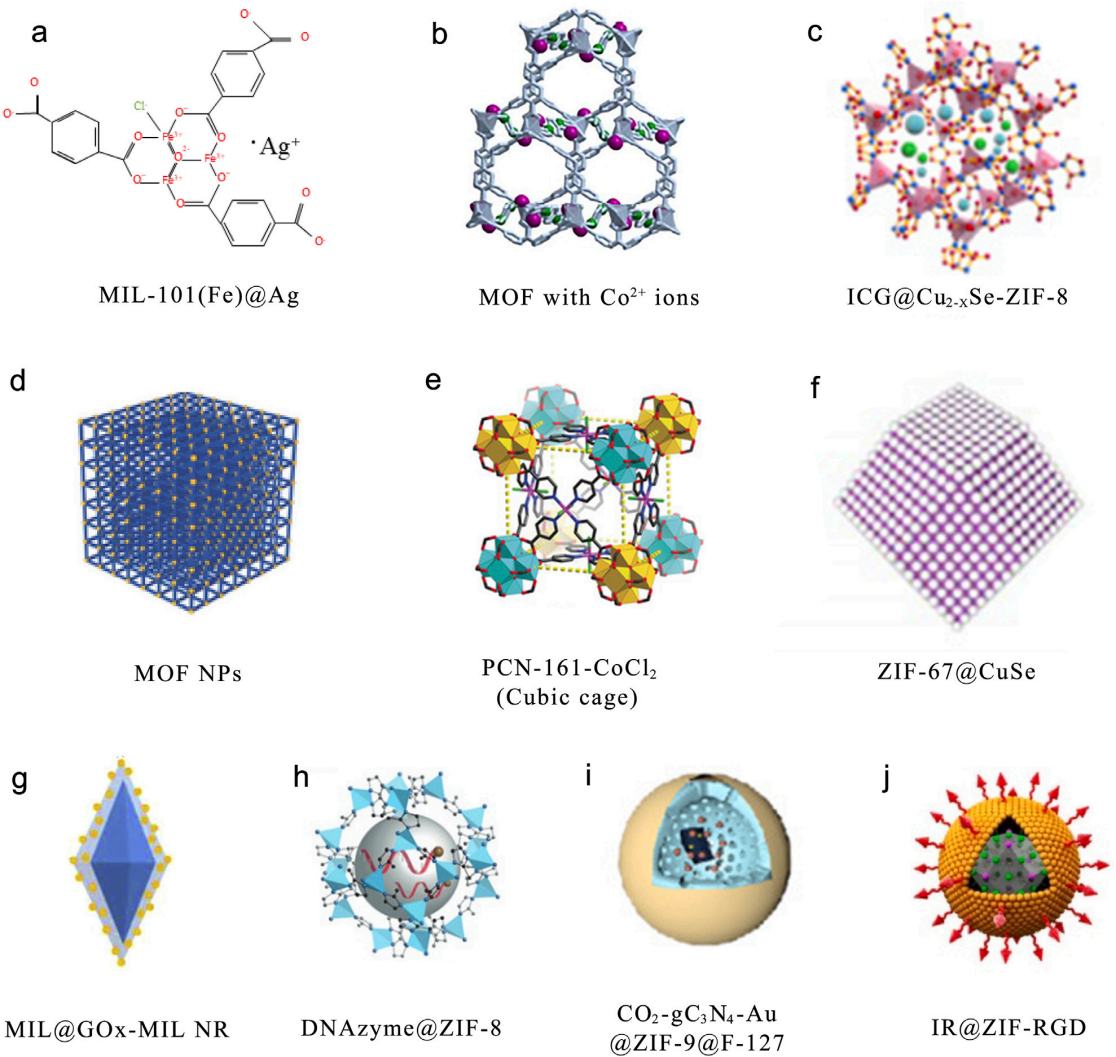
Some of the most commonly used MOFs. **(a)** Structurally modified Ag^+^-doped MOF MIL-101(Fe)@Ag derivative. Reproduced with permission (Li X. et al., 2022). Copyright 2022, the authors, distributed under the terms and conditions of the Creative Commons Attribution (CC BY) license. **(b)** Post-synthetic metal exchange is used to incorporate Co^2+^ into Zn-based MOF. Reproduced with permission (Hosseini et al., 2022). Copyright 2021, Elsevier. **(c)** A nanoplatform composed of Cu_2_-XSe and capped with zeolitic imidazolate framework-8 (ZIF-8). Reproduced with permission (Zou et al., 2022). Copyright 2022, Elsevier. **(d)** The modular synthesis of MOF NPs. Reproduced with permission (Wang S. et al., 2018). Copyright 2018, Wiley-VCH. **(e)** Zirconium(IV)-MOFs are transformed into Heterobimetallic MOFs containing Magnetic Anisotropic Cobalt(II) Centers. Reproduced with permission (Yuan et al., 2018). Copyright 2018, Wiley-VCH. **(f)** Synthesis of Monodisperse ZIF-67@CuSe@PVP Nanoparticles. Reproduced with permission (Wu et al., 2022b). Copyright 2021, American Chemical Society. **(g)** MIL@ glucose oxidase (GOx) -MIL NRs. Reproduced with permission (Li T. et al., 2020). Copyright 2020, Elsevier. **(h)** DNAzyme@ZIF-8 nanoplatform. Reproduced with permission (Wang et al., 2019). Copyright 2019, Wiley-VCH. **(i)** CO_2_-g–C_3_N_4_–Au@ZIF-8@F127 (CCAZF). Reproduced with permission (Xiao et al., 2021). Copyright 2021, Elsevier. **(j)** A nanocomplex depleted of adenosine triphosphate (IR@ZIF-RGD). Reproduced with permission (Yu et al., 2022). Copyright 2022, Elsevier.

MOFs represent a recently developed category of crystalline substances, distinguished by their periodic networks formed by connecting organic ligands to metal-containing nodes. These frameworks can be tailored to specific structural features, chemical compositions, synthesis technology of composite materials and functional applications by designing diverse topological architectures, representing a significant outcome of extensive exploration into highly porous materials (Chen et al., 2020a). Yaghi and colleagues synthesized the initial MOF in 1995, utilizing symmetrical porous organic molecules as the fundamental building blocks, with BTC as the organic ligand and Co^2+^ as the transition metal ion (Yaghi et al., 1995). By bonding and assembly with the metal centers (metal ions or metal clusters), they form metal-organic compound layers. The metal-organic compound layers, alternating with the layers whose composition is determined by the functionalization of the starting molecules, are referred to as the two-dimensional coordination compound structures of MOFs. MOFs are advantageous as drug carriers because of their unique crystalline structures, which offer extensive specific surface areas and significant porosity (Gao et al., 2022). They achieve high drug-loading efficiency while maintaining favorable biodegradability (Wen et al., 2021). Through the thoughtful design of inorganic and organic components, MOFs with specific architectures and functionalities can be developed. Additionally, the highly structured porous framework of MOFs holds significant promise for multifunctionality, making them perfect for various uses such as reaction catalysis, cell regeneration, and cancer treatment (Li S. et al., 2022; Gao et al., 2024; Gong et al., 2024). By using rare earth elements transition metals such as Co., Mn, and Fe as nodes (Lo Presti et al., 2023), MOFs are endowed with potential applications in catalysis (Zhang et al., 2019), combination therapy (Zhu et al., 2023), and sensing (Wang et al., 2023). Following the proposal of this concept, MOFs have rapidly advanced across multiple disciplines at a remarkable pace over the subsequent three decades. Recently, the capability of MOFs to integrate diverse functional materials, such as nanoparticles (NPs) (Li et al., 2020e; Sun, 2022; Wu et al., 2023), biological entities (Velásquez-Hernández et al., 2021), and composite materials has been continuously explored (Liang et al., 2021; Di Palma et al., 2022). The ongoing synthesis of multifunctional MOF heterostructures demonstrates greater advantages over individual components, as they are endowed with enhanced functionalities and novel properties (Liu et al., 2021). These MOF composites are organized and characterized by their structural classifications, chemical compositions, and functional applications, which are crucial for managing the expanding library of synthesized MOFs. These categories aid in examining the relationships between structure and properties, correlations between materials and applications, and interdependencies between composition and function. Furthermore, they enable the exploration of innovative architectures with targeted functionalities and guide the rational design and fabrication of MOFs tailored to specific applications. A range of techniques have been established to categorize the structural characteristics of MOFs; however, no unified comprehensive classification system exists that is universally applicable across diverse application scenarios. In this section, we will discuss several common classification strategies that provide insights into the arrangement of metal nodes, organic linkers, and void spaces within MOF frameworks, as well as interpretations of their functional behaviors and material properties. These classifications can be summarized in Figure 2.

### 2.1 Classification by structural features

#### 2.1.1 Topological classification

Network topology serves as a fundamental criterion for the classification of MOF structures. It delineates the arrangement and spatial configuration of metal nodes and organic linkers, thereby defining the architecture and geometry of the MOF structure. These topological characteristics are commonly depicted through graph-based representations known as networks or coordination networks. Each MOF structure is assigned a unique topological descriptor based on its underlying network, facilitating the systematic classification and comparison of diverse MOFs. MOF structures encompass MIL-101, characterized by simple cubic symmetry (Lin Z. et al., 2023), ZIF-8, which exhibits hexagonal close-packed symmetry (He et al., 2023), and more intricate configurations such as UiO-66. The latter integrates zirconium-based octahedral nodes and linear linkers (Yan et al., 2022; Iqbal et al., 2025).

#### 2.1.2 Categorization based on cages

Certain MOFs exhibit significant void spaces or cages within their structures. The term “cage” denotes a three-dimensional structural unit characterized by specific geometry and dimensions, formed through coordination bonds that connect metal nodes with organic linkers. These cage-like architectures generally possess considerable internal diameters, facilitating the encapsulation of guest molecules and offering spatial capacity for host-guest interactions. Precise regulation of the size and geometry of the cages can be accomplished through the modification of metal node types, organic linkers, and their connectivity patterns. For example, ZIFs commonly exhibit cubic or octahedral cages (Wang Y. et al., 2024), whereas metal-organic polyhedra are typically characterized by hexagonal prismatic and concave coordination cages (Wu X. et al., 2021).

### 2.2 Classification by chemical composition

#### 2.2.1 Classification by metal nodes

Within the framework of MOFs, metal nodes, which can be metal ions or clusters, bond with organic ligands through coordination to build periodic network structures. MOFs that incorporate transition metals such as Fe, Co., Ni, and Cu generally show high levels of catalytic activity and magnetic properties, which makes them suitable for applications in catalysis, magnetic materials, and sensing technologies (Zheng et al., 2023). Rare earth metal-based MOFs (e.g., La, Ce, Y) demonstrate unique optical and magnetic characteristics, making them ideal for luminescent materials and bioimaging (Xu et al., 2022). Metal nodes from the main group, such as Mg, Al, and Zn, typically exhibit excellent stability and biocompatibility, rendering them appropriate for drug delivery systems and medical applications (Stillman et al., 2023). It should be noted, however, that MOF-5 and the IRMOF family are Zn-based carboxylate materials. In early classic MOFs, the coordination interactions between metal nodes and organic ligands within their structural architectures are relatively weak (Gatou et al., 2023). They are prone to framework collapse in humid or aqueous environments, with extremely low hydrolytic stability (Yu Z. et al., 2021). This limits their applicability in scenarios requiring contact with water, such as water treatment and biomedicine, and hinders their applications in biomedicine, which is one of their core drawbacks. In addition, other well-studied biocompatible metal centers, such as Zr and Ti, Zr-based MOFs, with high stability and structural tunability as their core advantages, are suitable for thermocatalysis and applications in harsh environments; Ti-based MOFs, by virtue of their unique photochemical activity, are irreplaceable in the field of photocatalysis (Li J. et al., 2023), while also exhibiting excellent performance in biological antibacterial, bactericidal, anti-inflammatory, and osteogenic activities (Wang X. et al., 2022; Yan et al., 2022).

#### 2.2.2 Classification of organic ligands

MOFs containing biomolecules can be classified based on the types of organic linkers and biomolecules. The introduction of these molecules endows MOFs with capabilities such as molecular recognition, biosensing, biocatalysis, and self-assembly. The organic linkers in MOFs can incorporate a variety of biomolecules, including amino acids, peptides, proteins, nucleobases, carbohydrates, drugs, and porphyrins.

Amino acids (AAs) possess distinct advantages in ligand-based applications owing to their dual functionality, characterized by the presence of both carboxyl groups (-COOH) and amino groups (-NH_2_). Nevertheless, BioMOFs fabricated exclusively from amino acids remain relatively scarce; the majority are constructed by blending amino acids (or their modified derivatives) with organic ligands (Wang S. et al., 2021). The properties of peptide-based MOFs are contingent on the specific amino acid compositions utilized. Side chains not involved in metal coordination interactions can act as active sites. Peptides serve to bridge ligands, coordinate with metal ions, and form flexible porous materials endowed with the capacity for guest molecule adsorption (Cirujano et al., 2021). Proteins have complex structures and flexibility. Template-driven synthesis technology utilizes proteins as intelligent mediators to construct zeolitic MOF structures with the ability to encapsulate biomolecules, a property that underscores their prospective value in biocatalysis and biopharmaceutical applications (Wang H. et al., 2020).

Nucleobases possess a high self-assembly ability, metal-binding capacity, and numerous coordination sites (Beobide et al., 2015). By virtue of their structural rigidity, these properties result in the formation of cavities, thereby positioning them as optimal candidates for the design and fabrication of porous BioMOFs (Salama et al., 2022).

Carbohydrates have been effectively used as building blocks for self-assembled structures and act as efficient host molecules in BioMOFs. They have attracted much attention due to their biocompatibility and biodegradability. Among them, cyclodextrins, a type of naturally occurring cyclic oligosaccharides with a barrel-shaped cavity (Tian et al., 2020), have been used to prepare various types of CD-MOFs due to their characteristic of having a hydrophobic cavity and a hydrophilic surface (Li H. et al., 2020; Hu et al., 2021; Wei et al., 2021).

Drugs, as therapeutic agents, contain multiple ligand moieties within their molecular structures, thus numerous studies have reported the utilization of these molecules for constructing bio-metal-organic frameworks (Alves R. C. et al., 2021; Yang S. et al., 2022). Porphyrins consist of four pyrrole units linked by methine bridges (Ivanov and Boldyrev, 2014). This unique molecular architecture endows porphyrins with exceptional characteristics. Tetra(4-carboxyphenyl)porphyrin (H4TCPP), a commonly used porphyrin ligand, is extensively utilized in the fabrication of porphyrin-based MOFs, which in turn enables their broad application across diverse domains spanning gas storage, catalysis, and biotechnology (Jiang Q. et al., 2021; Zhao P. et al., 2021).

#### 2.2.3 Classification of reticular chemistry

Based on reticular chemistry, the classification of metal-organic frameworks (MOFs) primarily relies on net topology, building block connectivity, and structural complexity (Jiang H. et al., 2021).

Among these, edge-transitive nets serve as the most fundamental basis for classification, which are categorized into three types based on the uniqueness of their coordination figures: Type I nets possess unique coordination figures, corresponding to only one edge-transitive net, leading to a high success rate in design. For example, the 12-c fcu net (cuboctahedral configuration) (Rao et al., 2025). Type II nets have coordination figures shared by two to three nets, requiring regulation through details such as distortion angles and torsion angles. For instance, the 6-c pcu net (regular octahedral configuration) (Li J.-M. et al., 2021). Type III nets are composed solely of square or tetrahedral building blocks, with structures that are difficult to predict and require strict control over geometric parameters. Examples include square-configured nbo and lvt nets (Wang H. et al., 2018; Zhang Y. et al., 2018).

In the classification based on building blocks, connectivity is categorized according to the coordination number (n-c) of nodes and their combination modes, specifically uninodal nets (Lv et al., 2021), binodal nets (Guillerm and Eddaoudi, 2021), and multinodal nets (Guillerm et al., 2024). In terms of types, they include single-metal-ion-based, metal-cluster-based, and supermolecular building block (SBB)-based MOFs (Grancha et al., 2021).

### 2.3 Classification by synthesis technology of composite materials

MOFs can be incorporated with other base materials via various technical strategies to construct composite materials, where typical technical approaches encompass surface coating, electrospinning, 3D printing, and so forth.

The preparation of MOF-integrated composites as surface coatings mainly adopts *ex situ* synthesis strategies such as immersion coating. Specifically, by immersing the substrate material in a MOF precursor solution, MOF materials are allowed to deposit on the surface of the substrate or host matrix, forming a uniform coating. Methyl vanillate@ZIF-8 (MV@ZIF-8) is immobilized onto titanium surfaces to form a coating, mediated by polydopamine (PDA) (Si et al., 2023). This coating can sustainably release Zn^2+^ and MV, exerting antibacterial effects by inducing oxidative damage to bacteria, while promoting the early osteogenic differentiation of human bone marrow mesenchymal stem cells (hBMSCs) (enhancing alkaline phosphatase (ALP) expression and extracellular matrix mineralization). A bone microenvironment-responsive MOF coating is constructed on titanium surfaces. By releasing Ce^3+^ and Sr^2+^, it scavenges excessive ROS associated with osteoporosis (OP), restores mesenchymal stem cell (MSC) function, promotes new bone formation, and enhances implant-mediated fracture healing efficacy (Chen M. et al., 2022). ZIF-67 nanoparticles loaded with osteogenic growth peptide (OGP) are deposited as a coating on the TiO_2_ nanotube (TNT) surfaces of titanium implants (Tao et al., 2023). This coating not only possesses antibacterial properties and osseointegration capability but also effectively alleviates inflammatory responses and promotes immunomodulation by modulating macrophage polarization (reducing the secretion of pro-inflammatory cytokines).

MOFs can also form biocompatible osteoinductive materials with other materials via electrospinning. The microscale or nanofibrous networks constructed by electrospinning can closely mimic the structure of the extracellular matrix (ECM). Ghasemi et al. prepared poly-3-hydroxybutyrate-zein/UiO-66 electrospun composite scaffolds (Ghasemi et al., 2025). Cell viability, proliferation, adhesion, ALP activity, and ECM mineralization of the scaffolds were notably augmented, accompanied by significant upregulation of COLΙ, RUNX2, and OCN genes in MG-63 cells cultured on the scaffold surfaces. In addition, a β-cyclodextrin (β-CDs)/Ni-based MOF (β-CDs/Ni-based MOF) fibrous meshwork with intrinsic biocompatibility and biodegradability was produced through a highly efficient, fast, and controllable electrospinning technique, standing as a novel material option for orthopaedic uses (Lin J. et al., 2023). The β-CDs/Ni-based MOF scaffolds possess superior porosity, which can enhance proliferation as well as nutrient and oxygen transport, thereby facilitating more tissue regeneration.

With the continuous maturation of technology, 3D scaffolds with artificially controllable microstructures provide a potential strategy for bone tissue regeneration. For example, different amounts of UiO-66 nanocrystals were loaded onto alkali-heat treated 3D-printed titanium scaffolds, among which 1/2UiO-66/AHT exhibited excellent performance in osteogenesis and angiogenesis induction, and promoted intercellular communication by enhancing the paracrine effect (Liu et al., 2023). Xiao and his team prepared a novel magnetic nanocomposite, namely polycaprolactone (PCL)/Fe_3_O_4_@ZIF-8, via 3D printing technology (Xiao et al., 2024). This composite enhanced the proliferation and adhesion of rat bone marrow-derived mesenchymal stem cells (BMSCs); elevated the expression levels of osteogenesis-associated genes and proteins; stimulated the osteogenic differentiation of BMSCs via activation of the Wnt/β-catenin signaling pathway; alleviated infectious complications; and accelerated new bone formation in the context of infectious bone defects.

### 2.4 Classification by functional application

MOFs can be classified according to their functional applications, with particular emphasis on their intended uses across various domains. For instance, the presence of unsaturated metal sites within MOFs enables them to function as Lewis acid sites, thereby facilitating a range of chemical reactions (Rojas-Buzo et al., 2021). Furthermore, the porous structure and large surface area of MOFs endow them with superior performance in sensing and imaging applications. Among the MOF family, materials with structures from the MIL series are utilized. In particular, the combination of MILs with fluorescence has been employed for the detection of intracellular ATP molecules (Yao et al., 2021). Building on prior research findings, CD4^+^ and CD8^+^ helper T cells are acknowledged to participate in wound healing, hence immunosensors may serve as tools for monitoring the wound healing process (Neto et al., 2020; Solaimuthu et al., 2020).

Beyond these applications, MOFs are also used as drug carriers and vascular implants. MOF, with their high loading capacity and targeting ability, can control drug release. This is attributed to their high specific surface area, tunable structure, modifiability, and biodegradability, which make them efficient drug carriers (Hamedi et al., 2022; Aghazadeh Asl et al., 2023). Meanwhile, MOFs also show great potential in therapeutic interventions for diseases. As an illustration, the employment of copper-based MOFs in antithrombotic coatings for cardiovascular implant devices has seen growing adoption over the past few years (Sheng et al., 2024). With Cu-BTC employed as a catalyst, studies have verified that s-nitrosocysteine within blood samples undergoes transformation into cysteine and nitric oxide *in vitro* (Tsikas, 2021). Similarly, s-nitrosoglutathione may also undergo catalysis by more complex Cu-MOFs (Thai et al., 2022). Due to the nitric oxide-releasing properties of MOF/polymer composites, they are expected to be the preferred materials for new types of implants.

## 3 Preparation methods for MOFs with different physical properties

**FIGURE 3:**
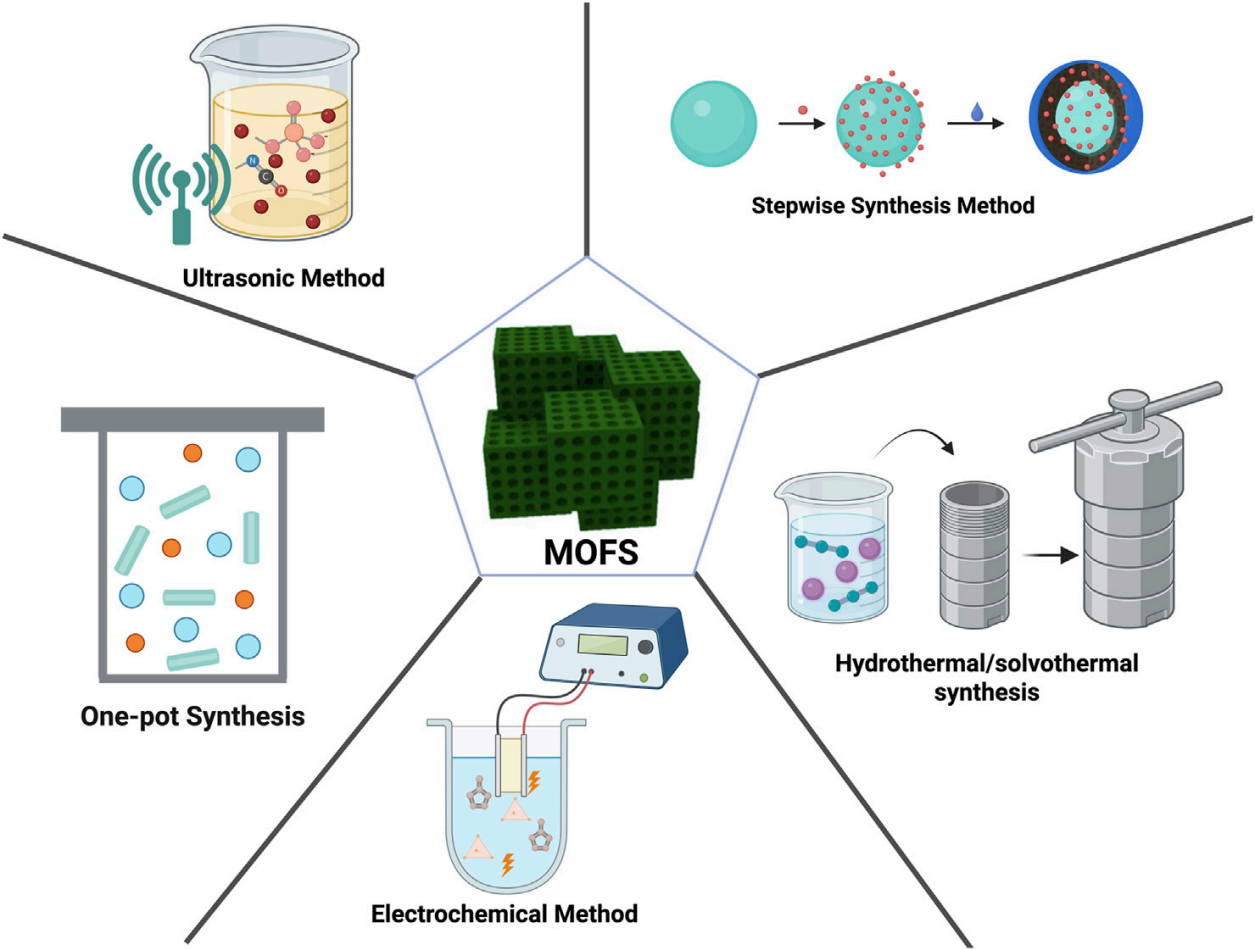
Schematic illustration of methods for preparing MOFs. The figure was created using Biorender.

Numerous methods exist for preparing MOFs. The application performance of MOF composites is closely related to their characteristic structures, as well as the apparent morphology and architecture of the composite materials. Even MOFs of the same type or identical composition can exhibit distinct properties when synthesized via different methods. This section presents various frequently employed MOF synthesis techniques: the one-pot method (solution precipitation), hydrothermal (solvothermal) synthesis, ultrasonic method, stepwise synthesis, electrochemical synthesis, and microwave-assisted synthesis (Figure 3). We summarize their advantages and limitations to guide researchers in selecting appropriate preparation strategies based on specific requirements.

### 3.1 One-pot synthesis (solution precipitation method)

The one-pot synthesis (solution precipitation) involves the co-mixing of precursors in a solvent followed by a simple precipitation reaction under stirring. ZIF-8 was synthesized by Beyer et al. through the mixing of 2-methylimidazole and a zinc salt solution at ambient temperature (Beyer et al., 2016). Huang et al. prepared Zn-based MOFs (MOCP) using Zn(NO_3_)_2_ and 1,4-benzenedicarboxylic acid (H_2_BDC) as reactants in a DMF solution containing triethylamine (TEA) at room temperature (Huang et al., 2003). This straightforward mixing approach allows for the quick synthesis of thermally stable and highly porous MOCP nanocrystals at room temperature in large amounts. The resulting MOCP materials exhibit high purity and yields (>90%).

The advantages of this method include low cost, high yield, and easily achievable experimental conditions. ZIF-8 can be modified to have bioactivity greater than that of standalone zinc ions (Shyngys et al., 2021). During precipitation reactions, insulin or nonsteroidal anti-inflammatory drugs (NSAIDs) can be incorporated into the precursor mixture, leading to their robust immobilization and encapsulation within ZIF-8 (Ho et al., 2020; Rohra et al., 2022). Researchers can control this process effectively by adding reactants at any point during the reaction. Nevertheless, the MOFs produced through synthesis frequently have impurities, rendering this approach inappropriate for uses that demand high purity.

### 3.2 Hydrothermal (solvothermal) synthesis

Creating materials through hydrothermal (solvothermal) synthesis is a technique that includes integrating solutions of metal ions and organic ligands as precursors in a closed system (e.g., a miniature autoclave), followed by heating in water or organic solvents to temperatures exceeding the solvent’s boiling point under autogenous pressure to facilitate the reaction. Chui et al. synthesized [Cu_3_(TMA)_2_(H_2_O)_3_]_n_ (denoted as HKUST-1) via a solvothermal reaction using Cu^2+^ and benzene-1,3,5-tricarboxylic acid (TMA) under elevated temperatures (Chui et al., 1999). HKUST-1 exhibits high porosity, enabling accessibility of its copper metal centers to solutes. This property allows Cu-BTC to catalyze the transformation of blood-borne S-nitrosothiols for sustained nitric oxide (NO) release (Yang L. et al., 2024). Li et al. employed Zn(NO_3_)_2_ and H_2_BDC as reactants in DMF to synthesize MOF-303 through solvothermal synthesis. Experimental data indicate that MOFs prepared by this method demonstrate superior stability, enhanced porosity, exceptional gas adsorption performance, and higher crystallinity compared to those synthesized via the one-pot approach (Li et al., 1999). This improvement is primarily attributed to the elevated pressure under hydrothermal conditions, which increases precursor solubility and thereby accelerates reaction kinetics and crystal growth. However, this method suffers from high costs, harsh reaction conditions, significant energy consumption, and limited controllability—reactants must be added entirely at once, and prolonged reaction times (hours to days) result in low efficiency, making it unsuitable for rapid large-scale synthesis. Extended durations may also lead to crystal overgrowth or undesired byproduct formation.

### 3.3 Ultrasonic method

Ultrasonic treatment utilizes the physical phenomena of cavitation effects, microjets, and localized environments with elevated temperature and pressure generated by ultrasound to enhance the mixing of dissolved reactants and crystal nucleation in solvents, thereby accelerating MOF synthesis. Yuan et al. first added specific quantities of iron (III) chloride, iron (II) chloride (at a Fe (III): Fe (II) molar ratio of 3:1), and 1 g of terephthalic acid (TPA) into a DMF solution (Yuan Z. et al., 2023). Following experimental design, cellulose fibers (CF) were incorporated at ratios of 20%, 30%, and 40% to prepare composite materials. The mixed solution was then subjected to ultrasonic treatment at 250 W power and 70 °C for 2 h. The interfacial regions between micro cavitation bubbles and the bulk solution exhibit extreme temperature/pressure changes and quick molecular dynamics (Qin et al., 2024), which facilitate the uniform growth of MIL-53(Fe) crystals on cellulose fiber surfaces and strengthen physical cross-linking and interactions involving hydrogen bonds between fibers and MOF crystals. This ultrasonic approach enables reactions that are challenging to achieve via conventional methods. For instance, reconstruction can be accomplished through mechanical disruption followed by re-ultrasonication.

Ultrasonic treatment constitutes an effective, environmentally sustainable, and streamlined methodology, particularly advantageous for laboratory-scale rapid synthesis and the fabrication of high-performance composite materials. Moreover, the acoustic cavitation effects facilitate specialized reactions that are not attainable through conventional techniques. Nevertheless, this approach encounters challenges related to equipment costs, material compatibility, process controllability, and scalability for industrial production.

### 3.4 Stepwise synthesis method

The stepwise synthesis method involves sequentially constructing the metal node and organic ligand networks. This approach enables precise control over MOF structure and functionality through post-synthetic ion exchange for metal doping, without compromising crystallinity (Fan et al., 2023). Cheng et al. developed CoCu-based bimetallic MOF nanoboxes (CoCu-MOF NBs) via a sequential cation and ligand exchange strategy (Cheng et al., 2021). TA-Co NBs were created by using tannic acid to etch Co-based ZIF-67. Subsequent cationic exchange in Cu^2+^ solution partially replaced Co sites with Cu atoms, forming TA-CoCu NBs. In the end, the TA ligands in TA-CoCu NBs were substituted with 2,3,6,7,10,11-hexahydroxytriphenylene (HHTP) ligands through ligand exchange, producing the final CoCu-MOF NBs.

The stepwise synthesis approach is highly effective in facilitating precise control over the composition and structure of materials, thereby substantially improving the catalytic performance of the oxygen evolution reaction. Nevertheless, the complexity of its synthetic procedures necessitates meticulous selection of ligand-metal combinations during exchange processes to avert framework collapse, consequently introducing an element of stochasticity (Fan et al., 2023). Furthermore, metal ions with diverse valences and ionic radii frequently adopt distinct coordination numbers and environments, thereby complicating the optimization of reaction conditions. Consequently, this often leads to reduced yields during the substitution of metal ions to replace the original metallic centers (Duan et al., 2020).

### 3.5 Electrochemical method

The electrochemical technique uses a metal electrode as the anode to emit metal ions through electrochemical oxidation or reduction occurring in a conductive medium, which subsequently interact with organic ligands in the solution to form monomers or various types of compounds and aggregates. This approach enables the construction of MOF thin films on electrode surfaces and has been widely adopted. Zhao et al. utilized a copper anode and 1,3,5-benzene tricarboxylic acid (BTC) as the ligand (Zhao T. et al., 2024). During the electrochemical reaction, the BTC ligands coordinated with copper ions to form an HKUST-1 thin film on the copper electrode. They observed that high-concentration electrolyte solutions provide abundant copper ions and BTC ligands, promoting rapid nucleation and growth of HKUST-1, resulting in large, uniformly distributed crystals. However, excessively high electrolyte concentrations may increase solution viscosity, hindering ion migration rates and compromising HKUST-1 growth quality. Similarly, high current densities accelerate electrochemical reaction rates and copper ion release, facilitating rapid nucleation and growth of HKUST-1 with uniform crystal distribution. Nevertheless, overly high current densities can elevate overpotential at the electrode surface, triggering side reactions that degrade the purity and performance of HKUST-1.

The electrochemical synthesis of MOFs offers advantages such as precise control over material composition and structure, mild reaction conditions, and enhanced electrical conductivity and electrochemical performance. Nonetheless, this technique is predominantly confined to the fabrication of thin films on conductive substrates, thereby limiting its widespread applicability.

### 3.6 Microwave-assisted method

The microwave-assisted method has been widely used for the rapid synthesis of MOFs under hydrothermal conditions. This approach employs microwave energy with frequencies spanning 300–300,000 MHz, offering an energy-saving and eco-friendly strategy for fabricating MOFs (Phan et al., 2023). Distinct from conventional heating techniques, microwave-assisted synthesis hinges on the interplay between mobile charges in polar solutions and microwave radiation to deliver the needed heat. It secures a consistent temperature increase throughout the reaction course and is not reliant on the demand for heat transfer within the reaction mixture, thereby accelerating crystal growth during MOF synthesis (Ren et al., 2022). The unique thermal properties of microwave-based approaches facilitate enhanced regulation of crystal size and structure, diminishing MOF particle dimensions down to the nanoscale range (Díaz de Greñu et al., 2021). This is crucial for the application of MOFs as drug delivery systems following different administration routes (Khan and Jhung, 2015). An appropriate particle size is a key factor ensuring the efficacy and safety of intravenous injection, as it is closely related to drug delivery efficiency, *in vivo* circulation time, organ/tissue accumulation, and targeting ability, among other aspects (Christodoulou et al., 2020; Wang A. et al., 2024). Similarly, when MOFs are administered via the pulmonary route, nanoparticles exhibit good colloidal stability, enabling localized drug delivery in the lungs without causing embolism due to excessively large particle sizes or rapid clearance due to excessively small ones (Fernández-Paz et al., 2020). However, it should be noted that MOFs require formulations with a size range of 1–5 μm. Particles within this range can penetrate deep into lung tissues through mechanisms such as sedimentation (in bronchioles) and Brownian motion (in alveoli); particles that are too large (>5 μm) tend to be retained in the trachea, while those that are too small (<1 μm) may be exhaled during respiration (Yu et al., 2025). Consequently, the microwave method has become an efficient way to synthesize MOFs.

While microwave-based approaches have established themselves as highly effective strategies for MOF synthesis, the microwave-assisted synthesis process is not devoid of constraints. The effectiveness of microwave heating is affected by the chosen materials, thereby necessitating meticulous regulation of synthesis parameters to optimize the characteristics of the end product. Moreover, microwave irradiation exhibits limitations such as challenges in reaction monitoring (Lozano Pérez et al., 2024).

## 4 Osteogenic mechanisms of MOFs

**FIGURE 4:**
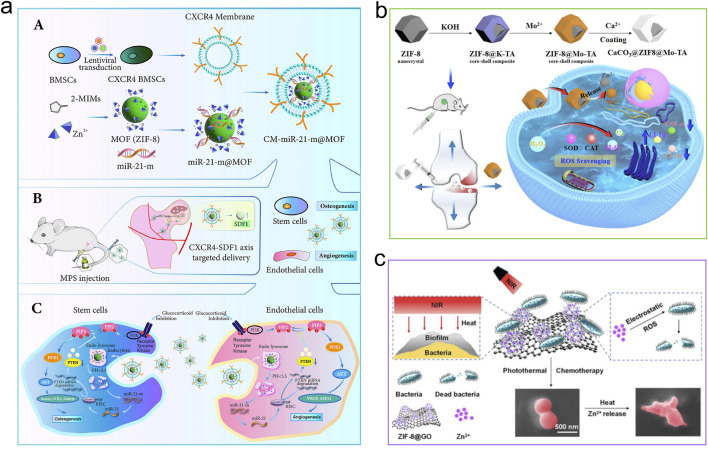
Mechanisms of MOFs for stimulating osteogenesis. **(a)** CM-miR-21-m@MOF release miR-21-m, activating the PI3K-AKT signaling pathway to regulate osteogenesis and angiogenesis. Reproduced with permission (Jiang et al., 2024). Copyright 2024, Acta Materialia Inc., published by Elsevier Ltd. **(b)** The compound CaCO_3_@ZIF@Mo-TA controls intracellular oxidative stress, eradicates excess free radicals, and generates free calcium ions that help repair and regenerate bone tissue associated with arthritis. Reproduced under the terms of the CC-BY license (Tan et al., 2025). Copyright 2025, The Author(s), published by Springer Nature. **(c)** Zn ions from ZIF-8 took the opportunity to react with the bacterial membrane, leading to its inactivation for bone repair. Reproduced with permission (Yang Y. et al., 2022). Copyright 2022, American Chemical Society.

MOFs are engineered through the systematic coordination of metal ions with organic ligands, allowing for precise modulation of their types and combinations to create various structures (Jiang H. et al., 2021). In biomedical applications, MOFs have garnered significant attention because of their ability to enhance bone growth, their distinct physicochemical characteristics like prolonged release of metal ions or therapeutic agents, photothermal responsiveness, pro-angiogenic activity, and anti-inflammatory effects (Shyngys et al., 2021; Lao et al., 2023; Li et al., 2024a; Li S. et al., 2024; Zheng et al., 2024). This section details the osteogenic mechanisms of MOFs (Figure 4), which can be categorized into the following sub-mechanisms.

### 4.1 Enhancement of physical characteristics of implants

The optimization of the physical properties of implants pertains to the structural customizability of MOF materials. This customization modifies interfacial properties to enhance variations in physical characteristics, including surface mechanical stress, growth interface topology, and surface charge, all of which are critically responsive to bone tissue interactions (Bolamperti et al., 2022; Wang L. et al., 2022). In recent years, the mechanisms of load transfer and the physicochemical properties of surfaces have been recognized as pivotal factors influencing interactions with diverse biomacromolecules and cells. These factors are crucial in attracting cells involved in osteogenesis at the implant interface and significantly impact their proliferation, differentiation, and mineralization processes (Wang L. et al., 2020; Shirazi et al., 2022). MOFs can be precisely engineered to replicate bone-like structures and elastic properties through strategic compositional modifications and optimized synthesis processes. These customized MOFs facilitate a more uniform distribution of mechanical loads at fracture sites, thereby preventing metallic implant components from assuming the role of primary load-bearing elements. This mechanical adaptation reduces the risk of stress-shielding, which can lead to bone atrophy and pathological remodeling (Luo et al., 2021; Raffa et al., 2021). Initially, Wang et al. applied a coating of Mg-MOF-74, a magnesium-based biocompatible compound, onto a 3D-printed porous Ti_6_Al_4_V substrate, subsequently encapsulating it with silk fibroin (Wang W. et al., 2022). Mechanical assessments revealed that the application of an Mg-MOF-74 layer to the porous titanium substrate significantly alleviated the stress-shielding effect attributed to stiffness mismatch, a prevalent problem in traditional titanium implants. This modification facilitated new bone formation within the titanium matrix, thereby improving osseointegration and achieving strong fracture fixation. Zhang and colleagues employed a biomimetic strategy, inspired by the microstructure of male eastern bluebird feathers, to develop a self-assembled MOF structure (Zhang et al., 2022a). This approach emulates the organic-inorganic interfacial configurations found in natural biomaterials, such as nacre, imparting the material with a distinctive non-iridescent structural coloration mechanism. The researchers augmented a supramolecular MOF that synergistically facilitates both antibacterial and osteogenic activities. Through the formation of Lewis acid-base adducts with 6-mercaptohexanoic acid nanoclusters and phytic acid (PA)-metal complexes, a multiscale supramolecular framework was established. This structure facilitates the recruitment of pre-osteoblasts and their subsequent differentiation into osteogenic cells. Phytic acid, abundant in phosphate groups, provides multiple sites for the chelation of Ca^2+^ ions, thereby expediting the process of biomineralization. Concurrently, coordinated metal ions such as Cu^2+^ and Zn^2+^ disrupt bacterial membrane integrity through sustained release, thereby inhibiting biofilm formation effectively (Zhang et al., 2023).

### 4.2 Metal ion release

In physiological environments, metal ion release is the process where metal ions detach and are emitted from the crystalline frameworks of MOFs. Various metal ions, such as zinc ions (Zn^2+^), magnesium ions (Mg^2+^), strontium ions (Sr^2+^), iron ions (Fe^3+^), and titanium ions (Ti^2+^), have attracted research attention due to their demonstrated potential in promoting the osteogenic differentiation of BMSCs and regulating the expression of osteogenesis-related genes in eukaryotic cells (Chen et al., 2017; Chen J. et al., 2022; Kang et al., 2022; Li et al., 2024a; Li S. et al., 2024; Zheng et al., 2024). Researchers have synthesized numerous osteogenic MOFs by incorporating the aforementioned metal ions into their crystalline frameworks, consistently finding that the sustained release of metal ions is a critical determinant of their superior osteogenic performance (Luo et al., 2024). As an illustration, Wang et al. developed an innovative bone-targeting orthopedic implant made of a nutrient element coating and polyetheretherketone (PEEK) (Wang H. et al., 2021). Through the incorporation of ZnO and Sr(OH)_2_ onto sulfonated PEEK surfaces (Zn&Sr-SPEEK), they demonstrated that the combined release of Zn^2+^ and Sr^2+^ from the coating eliminated harmful bacteria and greatly enhanced osteoblast activity in high glucose environments. Notably, the Zn&Sr-SPEEK implants demonstrated a strong capacity to restore high glucose-induced mitochondrial dyshomeostasis and dysfunction. This was accomplished by reducing the expression of the dynamin-related protein 1 gene, recovering mitochondrial membrane potential and clearing out ROS. As a result, osteoblast-mediated bone formation was significantly enhanced. Studies conducted on diabetic rat models with femoral/tibial defects at 4 and 8 weeks demonstrated that the nutrient element coating significantly enhanced bone restructuring and bone integration. In their study, Chen et al. developed zinc-based MOF films consisting of nanoscale and microscale ZIF-8 crystals on porous titanium surfaces through the application of hydrothermal and solvothermal processes (Chen et al., 2017). The ZIF-8 coatings promoted osteoblast proliferation and differentiation through sustained Zn^2+^ ion release. By upregulating the expression of osteogenesis-related genes like collagen type I (Col I), ALP, and bone morphogenetic protein (BMP), these ions facilitated the generation and calcification of the bone matrix. In a different study, Xiong and colleagues explored how low-intensity pulsed ultrasound (LIPUS) and Fe^3+^ together affect the proliferation and differentiation of osteoblasts (Xiong et al., 2024). Cell proliferation assays revealed that 400 μg/L Fe^3+^ exerted the strongest pro-osteogenic effect. ALP staining and mineralization assays demonstrated that LIPUS and Fe^3+^ synergistically enhanced osteoblast differentiation. Protein expression analyses further indicated that LIPUS and Fe^3+^ upregulated Wnt, β-catenin, and Runx2 signaling pathways, effectively promoting physiological bone regeneration and development.

Research has demonstrated that MOF can yield effective and sustained osteogenic outcomes through the continuous release of metal ions. This effect is primarily attributed to the increased ALP activity, enhanced mineralization of the extracellular matrix, and upregulation of osteogenic genes in MG63 cells. The release of metal ions serves as osteoinductive signals, while the simultaneous delivery of bisphosphonate linkers further augments bone mineralization (Li M. et al., 2022; Zhang et al., 2023). Moreover, Mg^2+^ ions generated via biodegradation have been observed to promote the osteogenic differentiation of MSCs by augmenting autophagic activity (Qi et al., 2021). Consequently, several hypotheses have been proposed concerning the mechanisms by which released metal ions facilitate osteogenesis. Some theories posit that these ions directly enhance the activity of key substances involved in osteogenic metabolism, whereas others propose that they stimulate gene expression to enhance the efficacy of bone repair.

It should also be noted that the controllable release of metal ions has been achieved through various strategies, with the core lying in leveraging the dynamic tunability and external stimulus responsiveness of MOF structures. Many MOFs undergo structural disintegration in acidic or alkaline environments, thereby releasing metal ions. Liu et al. discovered an easily designable resorbable guided bone regeneration membrane (PCL/DEX@Ca-Zol) based on drug-loaded metal-organic frameworks (Liu C. et al., 2024). Among them, the calcium ions, zoledronic acid, and dexamethasone embedded in the membrane can be specifically released in response at bone defect sites under acidic triggering, synergistically regulating the bone microenvironment (BME). Moreover, the strategy of using specific biomolecules (such as ATP) to trigger MOFs to release metal ions is also commonly employed in MOF design. Within the Mg/Zn metal-organic framework (MOF) synthesized by Yang et al., Zn^2+^ establishes a robust tetrahedral coordination complex via the nitrogen atoms in 2-methylimidazole (Xu Y. et al., 2024). Nevertheless, the adenine nitrogen moieties and phosphate groups of ATP are capable of forming chelates with zinc ions, and ATP exhibits a higher affinity for Zn^2+^ than 2-methylimidazole does. Within the periodontitis-specific inflammatory microenvironment marked by high ATP levels, this framework undergoes a response to ATP, facilitating the selective release of Mg^2+^ and Zn^2+^ at the inflammatory locus. Photoresponsive MOFs are also commonly used. For instance, RuFe-MOF undergoes structural changes under X-ray irradiation, releasing metal ions and generating ROS, which is applied in the synergistic radiotherapy and photodynamic therapy of tumors (Liu A. et al., 2025).

### 4.3 Drug loading

**FIGURE 5:**
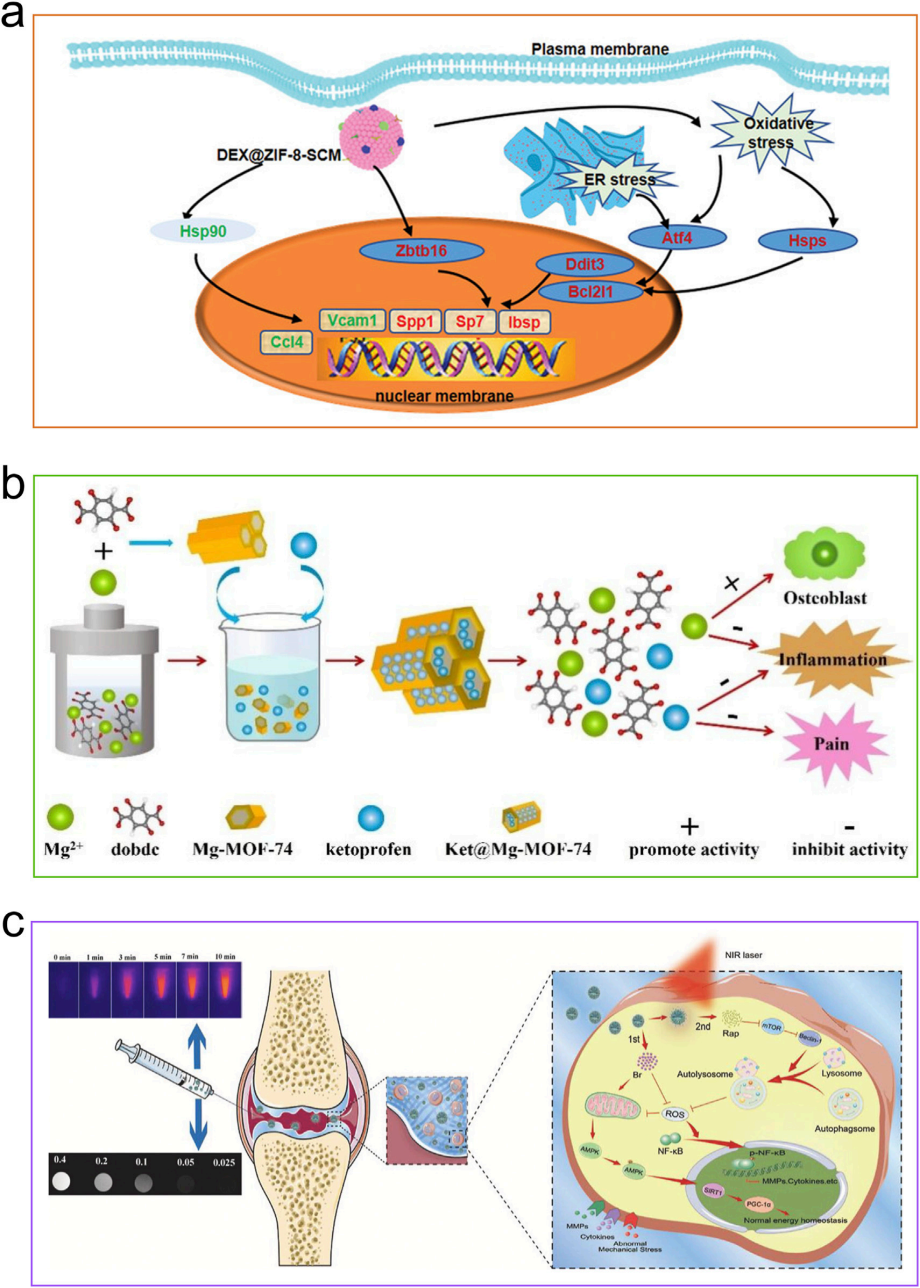
Applications of MOFs for delivering drugs. **(a)** DEX@ZIF-8-SCM nanocrystals exert a great role in promoting the osteogenesis of MSCs by the GR candidate. Reproduced with permission (Liang et al., 2022). Copyright 2022, Wiley-VCH. **(b)** Ketoprofen would be released from the channel of Ket@Mg-MOF-74 framework, inflammation suppressed and bone formation promoted. Reproduced with permission (Ge et al., 2021). Copyright 2021, Elsevier. **(c)** A system for delivering two drugs, featuring MPDA decorated with MOF. Reproduced under the terms of the CC-BY license (Xue S. et al., 2021). Copyright 2021, The Authors, published by Elsevier.

Thanks to their extensive surface area and significant porosity, MOFs are ideal for drug delivery (Figure 5). Therapeutic agents may be incorporated into MOFs via adsorption, encapsulation, non-covalent interactions, and covalent bonding. They enable effective delivery of short half-life pharmaceuticals, thereby enhancing treatment effectiveness. By customizing MOFs, they can display unique drug delivery traits like stimuli responsiveness and targeting, which allows for precise detection of pathological tissues (He et al., 2021). Osteoinductive agents encapsulated in MOFs effectively promote osteoblast differentiation and bone tissue regeneration through sustained release in physiological environments. At the same time, a strong blood supply provides nutrients and essential growth factors to osteoblasts, supporting synchronized and dynamic bone-forming activities in the bone microenvironment. In situations like inflammation, infection, or tumors, angiogenesis in specific areas is reduced, making it necessary to regulate externally by releasing pro-angiogenic agents. MOFs, with their extensive specific surface area, serve as excellent carriers for osteoinductive and angiogenic agents, where the drugs released effectively stimulate and hasten bone growth.

#### 4.3.1 MOF-mediated delivery of osteogenic inducers for enhanced bone formation

Liang and colleagues recently developed a system where stem cell membranes encapsulate dexamethasone-loaded ZIF-8, combining synthetic nanomaterials with natural plasma membranes (Liang et al., 2022). The MOF particles, coated with SCM, showed decreased immunogenicity and gained the capability to target BMSCs through homotypic binding, facilitating effective DEX delivery and DEX-induced bone repair. The porous nature of ZIF-8 combined with the natural targeting ability of SCM enabled DEX@ZIF-8-SCM to attain a high capacity for loading DEX, facilitating controlled release and enhancing targeted uptake by MSCs. Internalized DEX@ZIF-8-SCM exhibited high compatibility with cells and significantly boosted the osteogenic differentiation of MSCs. The RNA sequencing data unveiled the activation of the PI3K-Akt signaling pathway, leading to increased expression of transcription factors like Osterix and Smad4, which in turn promoted osteogenic differentiation in BMSCs. DEX@ZIF-8-SCM showed excellent compatibility with biological tissues and effectively stimulated bone regeneration in a bone defect model. By harnessing the synergistic interaction between zinc-based MOFs and raloxifene (Ral), Shen et al. produced multifunctional coatings for titanium implants (Shen et al., 2022). By integrating MOFs with Ral, localized drug delivery was achieved, and Ral’s hydrophobic groups were used to improve MOF stability in aqueous conditions. Clinically approved Ral, a benzothiophene derivative acting as a selective estrogen receptor modulator, boosts osteoprotegerin (OPG) production by activating the Wnt/β-catenin pathway in osteoblasts to fight against OP (Davis et al., 2020; Udagawa et al., 2021). The zinc-based MOF provides localized delivery of Ral and utilizes their synergy to establish a bone-promoting microenvironment around titanium implants by releasing Zn^2+^ ions, while also reducing Ral-induced bone loss in osteoporotic conditions. MOFs are capable of transporting specific signaling molecules to encourage osteogenesis, beyond just delivering osteoinductive drugs. Feng et al. loaded miRNA-5106 into ZIF-8 to achieve efficient cellular uptake and payload release at intracellular target sites (Feng et al., 2022). MiR-21, which promotes angiogenesis, and miR-5106, which supports osteogenesis, were chosen as model miRNAs and integrated into miR@ZIF-8 nanocomposites using a one-pot technique. According to the results, ZIF-8 carriers not only demonstrated high loading efficiency but also promoted cellular uptake and improved endosomal escape of miRNAs. Researchers conducted a systematic investigation into the therapeutic effects of miR@ZIF-8. Standard methods of delivering miRNA encounter challenges such as instability, poor penetration of cell membranes, and being prone to degradation by lysosomes. To address this, nano-sized ZIF-8 protects miRNAs through encapsulation and promotes their efficient cellular entry via endocytosis. In human umbilical vein endothelial cells (HUVECs) transfected with miR-21@ZIF-8, RNA sequencing analysis showed an upregulation of the MAPK and PID-HIF1-TF pathways, which in turn promoted angiogenesis. The study conducted by Yu et al. also found that miRNA-5106 was crucial in improving the healing of critical-sized bone defects *in vivo* through the activation of the Wnt/β-catenin and TGF-β/Smad pathways (Yu and Lei, 2021). Osteoinduction and the promotion of BMSC osteogenic differentiation were significantly influenced by the delivery of miR-5106 complexes.

#### 4.3.2 MOF-based drug delivery to promote angiogenesis

The short-lived nature of angiogenic agents when delivered externally constrains their multiple applications. By leveraging the delivery potential of MOFs, this limitation can be surpassed. Pathological conditions caused by reduced skeletal blood supply often lead to bone tissue necrosis (Zheng et al., 2022), which subsequently disrupts the osteogenic microenvironment, resulting in impaired bone formation and angiogenesis (Jiang et al., 2023). Deferoxamine (DFO) can lead to an increase in vascular endothelial growth factor (VEGF) (Zhao Y. et al., 2024). Even though DFO has a short plasma half-life, is rapidly cleared, and has poor biocompatibility, its local application continues to be a prevalent and effective method for promoting vascularization. In order to tackle these limitations, Li et al. utilized ZIF-8 as a carrier to increase the half-life of DFO (Li et al., 2023c). The promotion of vascularization by DFO@ZIF-8 nanoparticles was achieved by increasing the expression of type H vessels and vascular networks, while bone regeneration *in vivo* was facilitated by higher expression of osteocalcin (OCN) and BMP-2. RNA sequencing analysis indicated that DFO@ZIF-8 NPs caused an upregulation of the PI3K-Akt-MMP-2/9 and HIF-1α pathways in HUVECs, ultimately leading to neovascularization. Apart from supplying angiogenic agents, MOFs are capable of transporting specific signaling molecules to promote angiogenesis. A strategy mimicking biological processes was developed by Jiang et al. developed a strategy for the site-specific delivery of miR-21-m to necrotic femoral head lesions (Jiang et al., 2024). By coating BMSC membranes onto the surface of miR-21-m@MOF nanoparticles and further functionalizing them with membranes derived from CXCR4-overexpressing BMSCs (designated as CM-miR-21-m@MOF), these nanoparticles enhanced their targeting capacity for ischemic bony regions via the CXCR4-SDF-1 pathway (Zhang et al., 2022b). Bio-mimetic nanocomposites that mimic biological systems exhibited dual functionalities of targeting bony tissues and homing to ischemic areas concurrently. Mechanistic investigations further showed that miR-21-m delivery into target cells suppressed PTEN gene expression, thereby triggering the activation of the PI3K-Akt signaling pathway, which modulates osteogenesis and angiogenesis (Zhou et al., 2020).

Research suggests that the release of therapeutic agents can result in highly effective and durable bone repair. However, the metabolic processes of osteoinductive and angiogenic agents released from MOFs in physiological environments are not well understood, raising concerns regarding their biosafety.

However, it should be emphasized that in addition to the drug-loading approach, the ions released by the modified MOFs themselves can also stimulate angiogenesis. As a case in point, Si et al. validated via Transwell and tube formation assays using HUVECs that CuO@ZnO-coated titanium possesses superior angiogenic potential (Si et al., 2022). Released Cu^2+^ ions promoted angiogenic processes in HUVECs *in vitro* through the upregulation of vascular endothelial growth factor (VEGF) expression levels. Moreover, they also found that this composite material effectively promoted the adhesion and proliferation of hBMSCs, upregulated the expression of bone formation-related genes, and enhanced the mineralization ability of the extracellular matrix.

### 4.4 ROS scavenging

Under pathological conditions, elevated ROS levels induce substantial oxidative stress on bone tissue. This excessive ROS presence suppresses osteoblast activity while facilitating osteoclast formation, thereby disrupting the equilibrium of bone tissue. Such disruptions exacerbate local inflammation, accelerate bone resorption, and hinder bone regeneration (Tao et al., 2020; Yu et al., 2020). Consequently, the preservation of bone homeostasis is critically dependent on antioxidant activity. Strategies for scavenging exogenous ROS typically involve the neutralization of ROS through antioxidant agents, as well as their catalytic elimination via enzymatic or non-enzymatic pathways (Wang L. Y. et al., 2021; Kim and Kim, 2022).

MOFs are capable of delivering antioxidant agents and include metal atomic sites that can act catalytically. Different types of catalytic nanozymes have been designed, including those that act like catalase, superoxide dismutase, and glutathione peroxidase. Due to their adjustable active sites, structural variety, and outstanding biocompatibility, MOF-based nanozymes have wide-ranging potential applications in biomedicine and other fields (Xu Z. et al., 2024).

MOF-Fe is characterized by its unique unsaturated metal centers (UMCs). As a particle containing iron, it demonstrates properties similar to those of Fe_3_O_4_ and Fe_2_O_3_ particles. This distinctive characteristic imparts MOF-Fe with peroxidase-like activity, thereby positioning it as a potential catalyst (Cheng et al., 2023). Through the catalytic decomposition of hydrogen peroxide (H_2_O_2_), MOF-Fe effectively emulates the function of natural peroxidases. As a result, MOF-Fe holds significant potential for diverse applications as a peroxidase mimic (Thakur et al., 2021). In further investigations into the ROS-scavenging capacity of metal-organic framework iron (MOF-Fe), Xue et al. observed that experimental groups treated with MOF-Fe exhibited efficient scavenging of hydrogen peroxide, a component of ROS (Xue et al., 2024). The study further revealed that increasing concentrations of MOF particles were associated with a reduction in free radical levels. This conclusion was corroborated by DPPH radical scavenging assays, which demonstrated enhanced neutralization of DPPH radicals at higher concentrations of MOF particles. Notably, MOF-Fe particles were found to activate the bone morphogenetic protein (BMP) pathway by suppressing transferrin receptor 2 (TfR2), indicating significant potential for promoting bone formation.

Besides Fe-MOFs, various MOF-based nanozymes have been used for antioxidant purposes in bone tissue. Shu et al. developed Zn/Co-MOF-modified β-tricalcium phosphate (β-TCP) scaffolds to repair osteochondral defects. Shu and colleagues created β-tricalcium phosphate scaffolds modified with Zn/Co-MOF to mend osteochondral defects (Shu et al., 2023). Adjusting the concentration of the Zn/Co-MOF reactant solution allowed the MOF-TCP scaffolds to exhibit a broad spectrum of ROS scavenging properties and high biocompatibility. Remarkably, the MOF-TCP scaffolds advanced the osteogenic differentiation of BMSCs and the maturation of chondrocytes, while protecting them from oxidative stress by removing external ROS and supporting an anti-inflammatory microenvironment.

Simultaneously, Tan and colleagues engineered CaCO_3_@ZIF@Mo-TA, a compound exhibiting antioxidant properties, by employing ion-exchange and self-assembly techniques to mitigate oxidative stress in compromised cartilage (Tan et al., 2025). The pH-responsive microenvironment facilitates the degradation of calcium carbonate on the ZIF@Mo-TA surface, leading to the release of calcium ions that contribute to the repair and regeneration of bone tissue associated with arthritis. Experimental data indicate that the porous internal structure of ZIF@Mo-TA, characterized by its numerous active sites, substantially reduces the expression and accumulation of intracellular ROS. Moreover, it promotes the release of anti-inflammatory agents, collaboratively modulating intracellular oxidative stress levels and establishing a supportive immune microenvironment conducive to joint healing.

In another study, Liu et al. designed two cerium-based metal-organic frameworks with monovalent properties (Ce-MOFs): Ce (III)-BTC and Ce (IV)-BTC, for scavenging superoxide radicals (O_2_·^-^) and protection against ionizing radiation (Liu et al., 2022d). Both Ce-MOFs selectively reduce O_2_·^-^, serving as excellent superoxide dismutase (SOD) mimics. Similar to natural SOD and ceria nanozymes, the SOD-like catalytic mechanism of Ce-MOFs involves the redox cycling between Ce (IV) and Ce (III). Experiments conducted both *in vitro* and *in vivo* confirmed that the Ce (IV)-BTC nanozyme is effective at removing ROS.

Under physiological conditions, the osseous microenvironment sustains moderate levels of ROS. Pathological stimuli, such as infections and toxins, can exacerbate oxidative stress within bone tissue, leading to a deleterious cycle. The effective scavenging of excessive ROS can mitigate inflammatory responses and facilitate the repair of bone tissue. However, the overall efficacy of these interventions is limited, making them predominantly applicable as preventive measures in related research studies.

### 4.5 Antibacterial and anti-inflammatory

The vulnerability of bone tissue to infections and inflammation can significantly impede the bone formation process. Bacterial toxins and invasive enzymes have the potential to damage host cells, thereby exacerbating local inflammatory responses and disrupting bone development (Chen Z. Y. et al., 2021). In pathological states characterized by inflammation and infection, the upregulation of matrix metalloproteinases (MMPs) facilitates the degradation of cartilage tissue, while the inhibition of local angiogenesis contributes to bone loss (Mukherjee and Das, 2024). Consequently, it is imperative to employ effective antibacterial and anti-inflammatory strategies to maintain the osteoimmune environment. The subsequent section explores exogenous strategies for addressing bacterial infections and inflammation.

#### 4.5.1 MOFs for antibacterial applications

The enduring prevalence of bone infections, such as osteomyelitis, coupled with the limited efficacy of conventional treatments, presents formidable challenges within the field of orthopedics. The utilization of MOFs as delivery systems for antibacterial agents emerges as a straightforward and highly effective strategy for addressing bone infections (Zhang X. et al., 2022). MOFs are distinguished by their unique attributes, including adaptable pore structures, substantial specific surface area, and the capacity for surface customization. By mitigating the limitations associated with traditional antibacterial agents, MOFs exhibit significant potential for antimicrobial applications, attributed to their optimized topological configurations, exceptional durability, and superior thermal and chemical stability (Zhao et al., 2023).

Silver-based antibacterial agents exhibit high antimicrobial activity, broad-spectrum efficacy, and a low propensity to induce bacterial resistance (Calabrese et al., 2021). Silver ions (Ag^+^) possess the ability to eliminate bacteria through multiple mechanisms, including catalytic generation of ROS, prevention of biofilm formation, disruption of membrane integrity, and interference with bacterial metabolism (Huang et al., 2021). The team led by Wang designed a unique nanomaterial, small-sized Ag@MOF, which was integrated into sodium alginate (Alg) hydrogel to address periodontitis (Wang et al., 2025a). Ag@MOF managed to prevent the growth of *Escherichia coli* (*E. coli*) and *Staphylococcus aureus* (*S. aureus*) by employing different mechanisms such as interfering with bacterial metabolism, damaging membranes, and blocking biofilm formation (Bruna et al., 2021; Shinde et al., 2021; Ge et al., 2024). Once combined with Alg hydrogel, the composite enhanced endothelial cell growth and vascular formation, suppressed osteoclastogenesis, reduced inflammation in periodontitis (Zhu et al., 2024), prolonged retention time in the oral cavity, increased absorption, and d a reduction in the levels of pro-inflammatory cytokines (IL-1β, IL-6, TNF-α) (Popescu et al., 2021).

At the same time, Rauf and colleagues developed Zn-MOF nanoparticles loaded with ciprofloxacin and analyzed them using Fourier-transform infrared spectroscopy (FTIR), X-ray diffraction (XRD), and scanning electron microscopy (SEM) (Rauf et al., 2024). Zn-MOF@drug showed strong antibacterial effects on *Escherichia coli* and *Bacillus subtilis* due to the regulated release of metal ions and the drug, along with the combined effects of ciprofloxacin and zinc ions (Aden et al., 2023).

For the effective enhancement of implants’ antibacterial characteristics, the typical approach involves loading them with antibiotics and antimicrobial peptides. The effectiveness of antimicrobial peptides in clinical settings is hindered by both bacterial resistance and their expensive nature. The antimicrobial performance of implants can be improved by incorporating the right inorganic antibacterial agents. Yan and colleagues developed a multifunctional film made of fluorine-doped zirconium-based metal-organic framework (Zr-MOF) on titanium (Yan et al., 2022). Fumaric acid, acknowledged as a widely used antioxidant, served as the ligand for the Zr-MOF, while hydrofluoric acid functioned as a regulator of Zr-MOF film formation. The constructed fluorine-doped Zr-MOF film demonstrated superior biocompatibility and osteogenic potential, alongside robust antibacterial efficacy toward both Gram-positive *S. aureus* and Gram-negative *E. coli*. Furthermore, fluorine doping displaced fumaric acid within the framework, facilitating its liberation and thereby diminishing the stability of the Zr-MOF. The Zr-MOF liberated fumaric acid, which suppressed pro-inflammatory genes (NF-κB and IL-6) and upregulated the expression of the anti-inflammatory gene IL-4 in macrophages, manifesting potent anti-inflammatory properties.

Photodynamic therapy (PDT) and other emerging antibacterial therapies have achieved significant progress in recent years (Alves S. R. et al., 2021; Zeng et al., 2022). Phototherapy using MOFs can be accomplished by adding porphyrin derivative ligands or by loading photosensitizers/photothermal agents (Chen J. et al., 2021). For instance, Yang and colleagues developed a co-dispersed nanosystem with chemo-photothermal properties by growing ZIF-8 directly on graphene oxide (GO) nanosheets (Yang Y. et al., 2022). When exposed to a near-infrared (NIR) laser, GO generated localized heat around 50 °C, increasing the permeability of bacterial biofilms. Subsequently, Zn^2+^ released from ZIF-8 interacted with and disrupted bacterial membranes, enabling efficient sterilization at low temperatures. An inhibition rate of up to 85% against *E. coli* and *S. aureus* was demonstrated by this composite scaffold. Although PDT is a highly selective treatment that generates ROS with stronger bactericidal activity than organic antimicrobial agents (Pan C. et al., 2020; Hu et al., 2022), excessive ROS production under microenvironmental stimuli or antioxidant system dysregulation may cause cellular damage and pose risks to organismal health.

The effectiveness of PDT in clinical settings is restricted by its limited ability to penetrate deep into tissues and the risk of harming healthy cells with extended light exposure. Sonodynamic therapy (SDT), in contrast, employs ultrasound to stimulate sonosensitizers for ROS generation, enabling bacterial eradication under ultrasonic conditions. During ultrasound irradiation, cavitation effects occur, where rapidly collapsing bubbles generate intense shockwaves, localized high temperatures, high pressures, and hydroxyl radicals, significantly enhancing antibacterial efficacy (Pan X. et al., 2020). Yu et al. developed an ultrasound (US)-activated single-atom catalyst composed of gold nanorod (NR)-activated porphyrinic metal-organic frameworks (HNTM-Pt@Au) and red blood cell (RBC) membranes (Yu Y. et al., 2021). This system effectively treated methicillin-resistant *Staphylococcus aureus* (MRSA)-infected osteomyelitis under US irradiation, achieving a 99.9% antibacterial rate against MRSA after 15 min of ultrasound exposure.

#### 4.5.2 MOFs for anti-inflammatory applications

Delivering anti-inflammatory drugs through MOFs and releasing metal ions due to the disintegration of the framework is a direct method to alleviate inflammation in bone tissue. Considered a promising carrier, Mg-MOF-74 can deliver magnesium (Mg) and ketoprofen, the latter being a NSAID noted for its superior analgesic and anti-inflammatory qualities (Atzeni et al., 2021). The involvement of magnesium includes the adhesion, growth, and proliferation of osteoblasts, along with further mineralization of bones (Chang et al., 2020). Furthermore, it can curb inflammation by downregulating pro-inflammatory factors and promoting anti-inflammatory cytokines (Qiao et al., 2020). Ge et al. synthesized Ket@Mg-MOF-74 through post-synthetic modification, and tests demonstrated that this compound significantly reduced the expression of the cyclooxygenase-2 (COX-2), significantly upregulated the expression of osteoblast cytokines, and significantly downregulated the secretion of pro-inflammatory factors (Ge et al., 2021). According to Li et al., Mg/HCOOH−MOF was effectively synthesized from magnesium-based MOFs and was able to enhance the proliferation of MG63 cells with long-term use (Li et al., 2020d). According to qPCR results, this compound significantly modulates the expression of OCN, Axin 2, iNOS, and IL-1β, indicating its anti-inflammatory and bone-protective effects. Xue and colleagues developed a dual-drug delivery system using mesoporous polydopamine (MPDA) modified with MOFs, where rapamycin (Rap) was loaded into the mesopores and bilirubin (Br) was incorporated into the MOF shell layer (Xue S. et al., 2021). Rapamycin is recognized as one of the inhibitors of the mammalian target of rapamycin (mTOR), capable of inhibiting the proliferation of T lymphocytes (Lee et al., 2024). Br, a metabolite of bile acids, has been found to possess various biological functions, including the scavenging of ROS (Chen et al., 2020b). This dual-drug release system exhibits a good near-infrared laser-stimulated drug release effect, enabling bilirubin to scavenge cellular free radicals and rapamycin to enhance autophagic activity in cells. More importantly, the nanosystem enhances the energy metabolism of chondrocytes by engaging the AMPK-SIRT1-PGC-1α signaling pathway, which subsequently mitigates cell apoptosis *in vitro*.

Besides providing anti-inflammatory medications, endogenous signaling molecules are vital in triggering inflammation and the buildup of inflammatory factors. Zheng et al. developed a multifunctional nanoplatform IL-4-MOF@CaP, which significantly enhances functional bone regeneration *in vivo* (Zheng et al., 2020). IL-4 is the most potent activator of M2 macrophages, which reduce inflammation and aid in tissue repair by releasing anti-inflammatory cytokines like IL-10 and growth factors (Pan et al., 2025). Moreover, IL-4 binds to type I and type II IL-4 receptors, which activate the tyrosine kinases JAK1, JAK3, and TYK2, leading to the activation of STAT6. When STAT6 is activated, it triggers the expression of genes that reduce inflammation, such as M2 macrophage markers and anti-inflammatory cytokines (Allen, 2023). The platform successfully shields bioactive factors and discharges IL-4 in response to a low pH inflammatory environment, imitating the body’s natural inflammation resolution process. In addition, magnesium is provided by the platform for angiogenesis, gallic acid for ROS scavenging, and calcium and phosphate to contribute to the mineralization of the extracellular matrix in bones.

Antibiotics are effective in inhibiting bacterial colonization and proliferation. However, the widespread use of antibiotics has led to the emergence of multidrug-resistant pathogens, posing a significant threat to human health. While anti-inflammatory drugs can mitigate pain associated with pathological conditions, they do not prevent disease progression nor do they fundamentally address the underlying issue of impaired osteogenesis.

### 4.6 Synergistic therapy

Employing a combination of therapeutic approaches is a successful strategy to increase osteogenic efficiency. In recent years, numerous MOFs have achieved “1 + 1>2” effects by synergizing the bone-repair functions of their components. Osteoclasts play a pivotal role in bone tissue regeneration by coordinating bone resorption processes, which play a crucial role in bone remodeling and the subsequent creation of new bone tissue (Tsai et al., 2023). Researchers have created novel techniques to control osteoclast activity and enhance bone remodeling by utilizing the distinct characteristics of MOFs. Pang and colleagues enhanced the bone-targeting ability of immunostimulatory CpG-loaded MOF nanoparticles by modifying their surface with zoledronic acid (ZOL), a bisphosphonate approved by the FDA for anti-resorptive purposes (Pang et al., 2020). They observed that CpG oligonucleotides led to a partial inhibition of osteoclast formation and bone resorption, reducing them by about 50%. Significantly, the use of functionalized MOFs (immunostimulatory MOF (isMOF) and BT-isMOF) led to an over 80% reduction in osteoclast formation and entirely eliminated bone resorption activity.

An alternative strategy entails the development of MOF-integrated composite materials designed to modulate ambient pH conditions. These composites have the capability to regulate local pH levels, thereby creating an environment that mitigates excessive osteoclast activation, despite the fact that acidic conditions typically promote osteoclast activity and bone resorption (Jia et al., 2023). Additionally, by fine-tuning the mechanical properties of MOFs, it is possible to engineer a microenvironment that supports osteoclasts, thereby facilitating their physiological functions and contributing to bone remodeling (Wang L. et al., 2020). As a result, the synergistic osteogenic mechanisms demonstrate significantly enhanced bone-forming activity compared to any individual mechanistic component.

Synergistic therapy transcends a mere additive amalgamation of individual osteogenic mechanisms; it represents a complementary integration that leverages the strengths and mitigates the weaknesses of these mechanisms through mutual enhancement, thereby achieving superior osteogenic outcomes.

## 5 Applications of MOFs in different bone diseases

Up to now, comprehensive studies have been carried out to review the treatment applications and future possibilities of MOFs in various bone diseases. Based on the applications and corresponding therapeutic effects of MOFs in different pathologies, this section will highlight representative achievements of MOFs in orthopedic diseases, including OP, simple bone defects, bone defects with infections, bone defects related to diabetes, and tumor-associated bone defects.

### 5.1 Applications of MOFs in OP

OP is a common systemic metabolic disease characterized by decreased bone mineral density and bone mass, destruction of bone tissue microstructure, and increased bone fragility, resulting in an elevated risk of fractures.

However, the current conventional approach to treating OP is oral administration of anti-osteoporotic drugs, with drawbacks including first-pass metabolism and gastrointestinal side effects. Moreover, OP may result in microbial infections and necessitates the enhancement of angiogenesis to aid bone repair—such requirements are frequently unaddressed by conventional therapies. Furthermore, oral antibiotics carry the risk of inducing drug resistance during microbial infection treatment (Jiang et al., 2024).

In treating OP, possessing characteristics such as high specific surface area, high porosity, controllable degradability, and variable composition, MOFs not only serve as carriers for controlled drug release and exert multiple effects in treating OP and microbial infections through mechanisms like metal ion release—thus holding inherent advantages for the long-term treatment of OP—but also primarily function to regulate the bone microenvironment, stimulate the growth and differentiation of bone cells, and enhance bone density (Shi et al., 2025; Weng et al., 2024; Xu et al., 2025). For instance, certain MOFs are capable of being used as carriers for the regulated release of drugs (Alsaikhan et al., 2023), exert multi-functional roles in OP-related microbial infections through metal ion release (e.g., Mg^2+^) (Ge et al., 2021), and deliver bioactive factors (Li et al., 2020c).

Notably, Qin et al. developed an innovative hydrogel scaffold inspired by biological systems characterized by a soft-hard composite structure (Qin et al., 2025). This is different from MOFs that serve solely as drug delivery platforms. The structure includes a bilayer MOF, featuring ZIF-67 on the upper layer and ZIF-8 on the lower layer, produced using an *in situ* printing technique. This configuration enables spatiotemporal regulation of BMSC differentiation by controlling the release of Co^2+^ and Zn^2+^. The stimulation of osteogenic differentiation in BMSCs reduces OP symptoms, demonstrating their inherent benefits for treating OP (Li S. et al., 2024). *In vivo* experiments were conducted using New Zealand white rabbit models: Firstly, ZIFBH scaffolds (bilayer MOF hydrogels containing 15 mM ZIF-67 and 20 mM ZIF-8) and control scaffolds were subcutaneously implanted. After 2 and 4 weeks, the ZIFBH group showed less inflammatory cell infiltration, with significantly reduced inflammation at 4 weeks; no toxicity was observed in rabbit visceral organs, confirming its good biocompatibility. Secondly, different scaffolds were implanted in a 5 mm × 4 mm osteochondral defect model of rabbit knee joints. After 6 and 12 weeks, the ZIFBH group exhibited the best performance: at 12 weeks, the cartilage surface had minimal fibrosis and good integration with surrounding tissues, achieving the highest ICRS score. The subchondral bone volume fraction and bone mineral density were significantly increased. Immunohistochemistry showed high expression of cartilage-related proteins (ACAN, COL2A1) and osteogenic proteins (RUNX2), forming a complete bilayer repair structure with the highest O’Driscoll score, outperforming scaffolds with single MOF or no MOF.

### 5.2 MOFs utilization for basic bone defects

Bone defects caused by impairment of bone integrity can be divided into congenital deformities and acquired defects resulting from infection, trauma, or tumors. If not treated promptly, they may even lead to amputation (Wei et al., 2024). Therefore, patients bear significant physical, psychological, and economic burdens. Current bone grafting techniques are an effective clinical method for treating patients with large-area bone defects. Although this technique has saved many limbs at risk of amputation, it still has some obvious drawbacks. The main drawback is a prolonged healing period caused by poor osteogenesis and non-union at the bone junction, which may lead to many complications (Wang Q. et al., 2024). Managing these complications will further extend the duration of external fixation. It is well known that long-term wear of external fixators brings great inconvenience to patients, preventing most of them from resuming normal life within a year or even longer.

Simple bone defects have also seen extensive application of MOFs. For instance, Ahmed and colleagues developed UiO-66 nanomaterials that exhibited outstanding cytocompatibility and hemocompatibility, effectively enhancing osteoblast function *in vitro* (Sadek et al., 2022). Notably, UiO-66 implantation in defects not only demonstrated significant osteoid tissue formation and collagen deposition but also exhibited the potential to upregulate OCN and OPG expression *in vivo*. Xue et al. demonstrated that iron-based metal-organic framework (Fe-MOF) particles not only inhibit TfR2 but also act as biomimetic catalysts to scavenge hydrogen peroxide from ROS, showcasing their capability as bone regeneration agents. The functions of MOF-Fe included decreasing ROS levels and initiating the BMP signaling pathway (Xue et al., 2024). Wu et al. synthesized bio-MOF-1 coatings on alkali-heat-treated titanium at varying concentrations and systematically evaluated their cytocompatibility and bone development effectiveness in both experimental and natural conditions (Wu et al., 2022a). As a representative bio-friendly MOF with a biologically derived structure, bio-MOF-1 owes its properties to a zinc core and adenine ligands. Exhibiting remarkable thermal stability and biocompatibility, the coating also provided a prolonged release of Zn^2+^, boosting the expression of osteogenesis-associated genes and proteins. In addition, with titanium as the scaffold, the bio-MOF-1 coating on titanium implants substantially improved early osseointegration where the bone meets the implant.

It is noteworthy that in current studies on treating bone defects using MOFs, the bone regeneration efficacy of MOF-based materials has been investigated through animal bone defect models; however, these studies each have distinct focuses and certain limitations. Zhang and his team focused on L-Asp-Cu(II) bio-MOF. In a 5 mm-diameter calvarial defect model of SD rats, surgical implantation of 30 μg/mL L-Asp-Cu(II) significantly increased bone volume fraction (BV/TV) and bone mineral density (BMD) at 8 weeks, and activated the TGF-β/BMP pathway to promote vascularized bone regeneration (Zhang Y. et al., 2025). However, long-term safety and validation in large animal models remain unaddressed. Yu et al. developed a Zn-MOF composite 3D-printed scaffold. In a 3 mm × 4 mm femoral condyle defect model of rats, the PHCZ group achieved a bone volume fraction of 31.39% ± 3.04% at 12 weeks, significantly higher than the control group, and exerted anti-inflammatory-osteogenic synergistic effects by regulating macrophage polarization (Han et al., 2025). Nevertheless, the toxicity risk of long-term Zn^2+^ release and the specific mechanism of MCF require further clarification. Sun et al. targeted aged rat calvarial defects (6 mm diameter). Mg-Ce-MOF scaffolds combined with SKL2001 improved the senescent microenvironment by activating the Nrf2 pathway, with new bone area reaching 42.29% ± 1.87% at 12 weeks (Sun et al., 2025). However, the synergistic mechanism between SKL2001 and MOF, as well as the insufficient sample size of aged models, need further research. However, it is important to emphasize that the significance of their present research lies in modulating the senescent microenvironment (SME) to retard the senescence process of BMSCs, thus offering a viable strategy to facilitate the repair of age-related bone defects. This constitutes a highly intriguing strategy.

### 5.3 Applications of MOFs in infected bone defects

**FIGURE 6:**
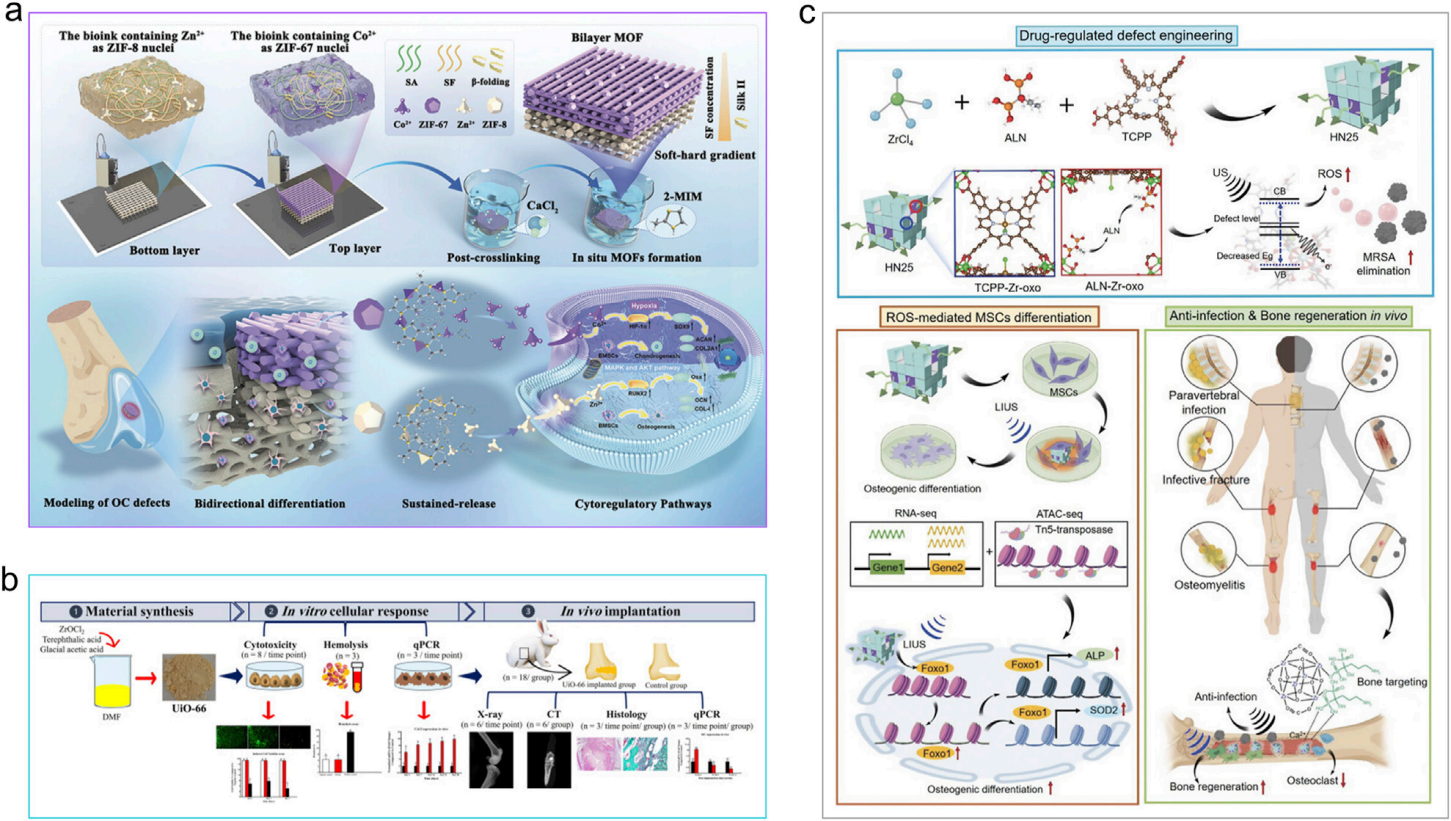
Applications of MOFs for Simple and Infected Bone Defects. **(a)** ZIF-8 releases Zn^2+^, which enhances osteoblast differentiation by RUNX2—a critical regulator of osteogenesis. Reproduced with permission (Qin et al., 2025). Copyright 2024, Wiley-VCH. **(b)** The use of UiO-66 nanomaterial in implants promotes the recovery of critical-sized bone defects. Reproduced under the terms of the CC-BY license (Sadek et al., 2022). Copyright 2022, The Author(s), Springer Nature. **(c)** ALN is employed to create a porphyrin-based MOF sonosensitizer (HN25) with enhanced antibacterial properties responsive to ultrasound. Reproduced with permission (Ma et al., 2024). Copyright 2023, Wiley-VCH.

MOFs are essential in managing infected bone defects through three primary mechanisms: delivering antimicrobial drugs, leveraging their intrinsic antibacterial properties, and modulating the bone microenvironment (Figure 6). The team led by Ma engineered a sonosensitizer with a defective MOF mediated by alendronate (ALN) (Ma et al., 2024). HN25 increases the accessibility of chromatin for genes related to osteogenesis, such as FOXO1, and aids in bone repair through low levels of ROS when exposed to low-power ultrasound. Rapid clearance of methicillin-resistant MRSA, suppression of osteoclast activity, and enhancement of bone regeneration and differentiation are achieved by this system. In their study, Liu Y. et al. (2025) created a gallium-based MOF (GaMOF) coated with quaternized chitosan (QCS) that carries a positive charge, serving as a “capture agent” to ensnare MRSA by disrupting the TCA cycle (Liu Y. et al., 2025). A radially porous crystal gel embedded with Me and QCSGaMOF was additionally fabricated by them using directional solidification. The oriented porous structure enhances osteointegration by guiding osteoblast ingrowth. Tan et al. synthesized MgCu-MOF-74 nanoparticles with varying Cu content via a one-step hydrothermal method (Liu J. et al., 2022). MgCu-MOF-74 enhances the viability of human osteosarcoma cells (SaOS-2), ALP levels, collagen synthesis, and osteogenic gene expression. Additionally, Cu^2+^-doped samples exhibit heightened sensitivity to acidic microenvironments produced by bacteria, demonstrating stronger antibacterial activity compared to Mg-MOF-74.

MOF-decorated 3D-printed scaffolds have excellent therapeutic advantages for severely defective bones. They not only exert the multiple osteogenic properties of MOFs in osteogenesis but also utilize the scaffolds to avoid the issue that MOFs, being too small at the nanoscale, cannot connect the broken ends of the defect. Zhu and her team focus on Cu-MOF-74-decorated 3D-printed PCL/HAp composite scaffolds, assessing their antibacterial and osteogenic potential in bone defect repair via *in vitro* experiments (Zhu et al., 2025). Key findings highlight that Cu-MOF-74 concentrations of 0.05%–0.2% balance dual functionalities: the 1% concentration achieves 90.07% and 80.03% inhibition rates against *Staphylococcus aureus* and *Escherichia coli*, respectively; low concentrations (≤0.2%) promote the proliferation of rat BMSCs; the 0.05% group shows the highest calcium deposition and osteopontin (OPN) expression; and the 0.2% group exhibits optimal ALP activity. Additionally, Cu^2+^ release follows a biphasic pattern—an initial burst within the first 7 days, followed by sustained release up to 28 days.

Notably, the study has limitations: no *in vivo* animal experiments were performed to validate efficacy; high concentrations (≥0.5%) of Cu-MOF-74 induce cytotoxicity; and long-term properties of the scaffolds, such as *in vivo* degradation kinetics and mechanical stability, remain uninvestigated.

### 5.4 Applications of MOFs in diabetic bone defects

Hyperglycemia induces excessive production of ROS in intracellular mitochondria. These ROS directly disrupt the osteogenic microenvironment, interfere with the bone healing process, lead to poor healing of bone defects, and severely impair patients’ quality of life (Khosla et al., 2021; Li X. et al., 2021).

**FIGURE 7:**
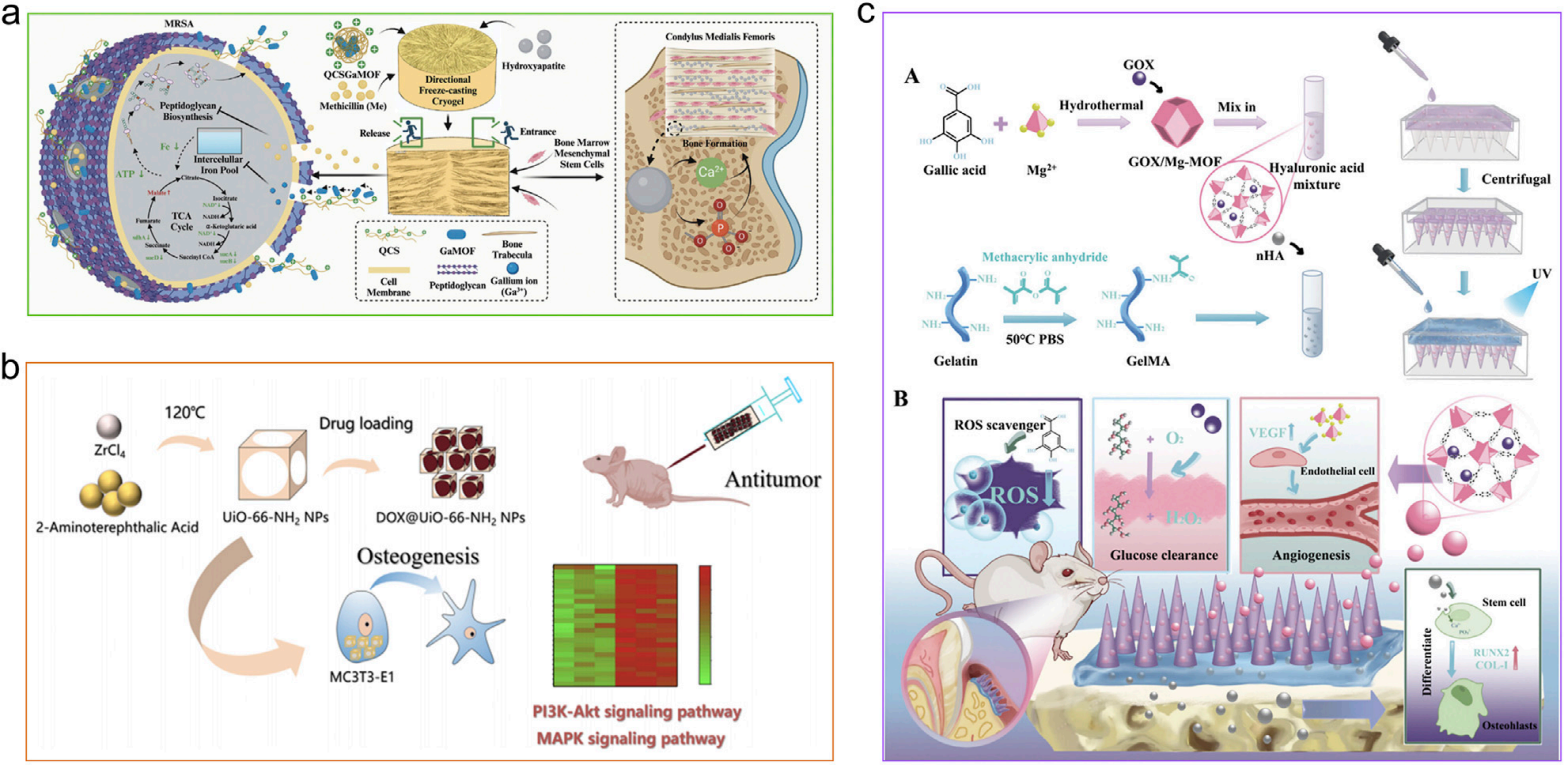
The application of MOFs includes antibacterial, antitumor, and diabetes treatments. **(a)** Me/QCSGaMOF@Cryogel reactivating methicillin effects and guided bone formation. Reproduced with permission (Liu Y. et al., 2025). Copyright 2025, Wiley-VCH. **(b)** UiO-66-NH_2_ NPs, which are zirconium-based MOF nanoparticles with amino functionalities, are used as dual-purpose nanomaterials for bone tumor therapy and stimulating osteogenesis. Reproduced with permission (Yuan J. et al., 2023). Copyright 2023, American Chemical Society. **(c)** Mg-MOF released from d-MNs modulates the local microenvironment in soft tissue regions by reducing ROS, lowering blood glucose, and releasing magnesium ions to promote angiogenesis. Reproduced with permission (Qu et al., 2024). Copyright 2025, Wiley-VCH.

By modulating pathological conditions such as hyperglycemia, oxidative stress, and inflammation, MOFs play a crucial role in the repair of diabetic bone defects, thereby promoting bone regeneration and angiogenesis (Figure 7).

These studies illustrate the extensive potential of MOFs in the context of diabetic bone regeneration, providing innovative strategies for future research and clinical applications. In this regard, Liu et al. developed a Mn@Co_3_O_4_@Pt nanozyme, designated as MCPtA, which was synthesized from a MOF loaded with alendronate (ALN) and Mg^2+^ (Liu S. et al., 2025). The substitution of Mn atoms into the Co_3_O_4_ nanocrystal structure modulated the electronic configuration, resulting in a significant enhancement of SOD/CAT catalytic activity. The incorporation of Pt nanoparticles, which mimic glucose oxidase (GOx), enabled MCPtA to effectively perform multi-cascade catalysis, facilitating the clearance of glucose and ROS. This process contributed to the regulation of hyperglycemic conditions and restored the balance between osteoblasts and osteoclasts. By inhibiting inflammatory responses induced by glucose-ROS and promoting the development of bone and vascular tissues, the nanozyme composite hydrogel significantly improved bone regeneration in diabetic patients. Fan et al. introduced a multifunctional bilayer microneedle (d-MNs) system for hard tissue repair. The d-MN matrix is composed of gelatin methacryloyl (GelMA) infused with nano-hydroxyapatite (nHA), which supports osteoblast differentiation and alveolar bone regeneration (Qu et al., 2024). Soft tissue repair is facilitated by a d-MN tip constructed from hyaluronic acid (HA) integrated with a glucose oxidase (GOX)-loaded magnesium Mg-MOF, which imparts both hypoglycemic and anti-inflammatory properties. This dual-functional design ensures a comprehensive therapeutic approach, encompassing hypoglycemic effects, anti-inflammatory responses, neovascularization, and osteogenesis.

Severe tissue dysfunction in diabetic patients represents a tough clinical challenge. Both bacterial infections and chronic inflammation drive disturbances in the diabetic microenvironment and impaired tissue regeneration, thus, there is a greater need for MOFs with composite therapeutic mechanisms. Zhang et al. evaluated the *in vivo* efficacy of Gelma@Sr-ZIF-8 hydrogel using two diabetic rat models: In the diabetic rat skin infection model (15 mm-diameter infected wounds), local injection of 2wt% Sr-ZIF-8 (Sr/Zn molar ratio 20/100) hydrogel followed by UV curing resulted in a 97% wound healing rate at 14 days, with significantly higher bacterial clearance than the control groups, accompanied by enhanced collagen deposition and angiogenesis. In the diabetic rat femoral defect model (3 mm × 4 mm), 8 weeks after implantation of the hydrogel, the bone volume fraction (BV/TV) reached 38.6% ± 3.2%, significantly higher than the control groups, while promoting M2 macrophage polarization and high expression of osteogenic markers (Runx-2, OCN). Its core advantage lies in the synergistic effects of Zn^2+^ and Sr^2+^ in exerting antibacterial, anti-inflammatory, and bone regeneration-promoting functions with good biosafety (Zhang S. et al., 2025). However, there are limitations: it is only based on rat models without large animal validation; the potential cumulative effects of long-term ion release are unclear; and its repair effect on complex conditions such as diabetes with neuropathy has not been explored.

The studies underscore the substantial potential of MOFs in the context of diabetic bone regeneration, thereby presenting novel strategies for future research and clinical application.

### 5.5 Applications of MOFs in bone tumor bone defects

Currently, the primary approach to treating bone tumors is surgical excision, although it often causes local bone defects and tumor recurrence. As a result, it is important to develop biomaterials that can simultaneously address tumor treatment and bone healing after surgery. MOFs, with their unique porous structure, hold significant potential in regenerative medicine and drug delivery. Leveraging their biocompatibility and biodegradability, MOFs can act as drug carriers or sustained-release systems for bioactive factors to promote the renewal and mending of bones. The team led by Qu developed a multifunctional MOF-modified injectable calcium phosphate cement, integrating cobalt-coordinated tetrakis(4-carboxyphenyl) porphyrin (Co-TCPP) (Qu et al., 2021). The inclusion of Co-TCPP not only kept the cement’s superb injectability but also decreased the setting time, boosted compressive strength, and provided the cement with outstanding photothermal capabilities for efficient tumor therapy. This system overcomes the limitation of traditional calcium phosphate cement (CPC), which fails to eliminate residual tumor cells post-surgery, while simultaneously promoting bone and vascular regeneration *in vivo*, demonstrating ideal osteogenic and angiogenic capabilities. In their study on postoperative therapy, MOF composite scaffolds are also frequently used to meet the requirements of various therapeutic needs. Zeng and colleagues developed a chitosan composite scaffold (CS/DOX@Ti-MOF) aimed at treating tumors and repairing bones (Zeng et al., 2024). The amino-functionalized titanium-based metal-organic framework (NH_2_-MIL-125(Ti), Ti-MOF) exhibits a high specific surface area (1,116 m^2^/g) and excellent biocompatibility, enabling the loading of doxorubicin (DOX), a chemotherapeutic agent while promoting osteogenic differentiation. The composite scaffold demonstrates improved physical mechanical properties and a rough surface, facilitating cell adhesion. In the tumor microenvironment, the scaffold releases DOX in a responsive manner to destroy residual tumor cells and then provides areas for cell attachment, growth, and differentiation. This dual functionality enhances bone repair and achieves adjunctive therapy for postoperative bone tumors. In their study, Yuan J. et al. (2023) focused on the interaction between DOX and MOFs for bone tumor therapy, designing amino-functionalized zirconium MOF nanoparticles containing DOX (DOX@UiO-66-NH_2_ NPs). Lung injury was significantly reduced by DOX@UiO-66-NH_2_ NPs *in vivo* compared to free DOX. Notably, internalized UiO-66-NH_2_ NPs markedly activated the PI3K-Akt and MAPK signaling pathways, promoting osteogenic differentiation of pre-osteoblasts. UiO-66-NH_2_ nanoparticles are positioned as a multifunctional nanomaterial for both bone tumor treatment and the enhancement of bone growth.

## 6 Advantages of MOFs over other types of nanoparticles used in bone regeneration

Within the domain of bone regeneration, MOFs, as an emerging class of nanocarriers, demonstrate notable superiority over other nanoparticle categories including inorganic nanoparticles (e.g., hydroxyapatite (HA), gold nanoparticles (AuNPs), iron oxide nanoparticles (IONPs), mesoporous silica nanoparticles (MSN)) and organic nanoparticles (e.g., liposomes, micelles, chitosan (CS), poly(lactic-co-glycolic acid) (PLGA)).

### 6.1 Higher drug loading capacity and controllable release performance

MOFs feature ultra-high specific surface areas and adjustable porous architectures (e.g., ZIF-8 with a specific surface area reaching 1700 m^2^/g), facilitating high-efficiency loading of diverse therapeutic agents (including antibiotics, growth factors, and metal ions). Their drug-loading capability exhibits marked superiority over that of conventional carriers such as chitosan and MSN. The pore dimensions and topological structures of MOFs are amenable to precise modulation, preventing abrupt drug release and thereby enabling sustained and controlled release profiles (Xu H. et al., 2024). For instance, ZIF-8 can undergo pH-responsive degradation (structural disintegration in acidic microenvironments) to slowly release loaded bisphosphonates or zinc ions, matching the long-term requirements of bone regeneration (Yun et al., 2025). In contrast, the release rate of polymer nanoparticles such as PLGA is difficult to precisely control, prone to early burst release or insufficient late-stage release.

### 6.2 Multifunctional synergistic effects

MOFs are capable of exerting diverse biological functionalities concurrently via the integration of metal ions and organic ligands, whereas most traditional nanoparticles have relatively single functions.

#### 6.2.1 Metal ion release

Metal ions such as zinc (Zn^2+^), magnesium (Mg^2+^), and calcium (Ca^2+^) released during MOF degradation can directly promote osteogenic differentiation (e.g., Zn^2+^ upregulates the expression of osteogenic-related genes such as RUNX2 and ALP) and angiogenesis, while inhibiting excessive osteoclast activation (e.g., by regulating the RANKL/OPG ratio) (Liu et al., 2022e). For example, Zn^2+^ released from ZIF-8 has both osteogenic and antibacterial effects, whereas HA mainly focuses on osteogenesis, and silver nanoparticles (AgNPs) are only antibacterial (Eivazzadeh-Keihan et al., 2020).

#### 6.2.2 Carrier-drug synergy

MOFs can simultaneously load antibiotics (e.g., vancomycin) and growth factors (e.g., BMP-2) to achieve “antibacterial-osteogenic” synergistic therapy (Feng et al., 2022). Although MSN can also load multiple drugs, it is inferior to MOFs in terms of loading diversity and synergistic regulation.

### 6.3 Excellent biocompatibility and biodegradability

MOFs can significantly reduce toxicity risks by selecting endogenous metal ions (e.g., Zn^2+^, Mg^2+^, Ca^2+^) and biodegradable organic ligands (e.g., imidazole, carboxylic acid). In addition, the degradation rate of MOFs can be adjusted by ligand stability (e.g., controlling the degradation rate by changing ligand chain length), matching the dynamic balance of “material degradation-new bone formation” during bone regeneration (Farjaminejad et al., 2024). In contrast, inorganic nanoparticles such as HA degrade slowly and tend to remain for a long time.

### 6.4 Easy functional modification and targeting

MOFs are rich in active functional groups (e.g., amino, carboxyl) on their surfaces, allowing convenient targeted modification (e.g., conjugation with bisphosphonates or bone-targeting peptides) to enhance specific accumulation at bone defect sites (Li et al., 2024a). For example, ZOL-modified ZIF-8 nanoparticles can achieve bone-targeted delivery through high affinity with hydroxyapatite in the bone matrix, reducing side effects on other tissues (Choi et al., 2023). In contrast, targeted modification of traditional nanoparticles (e.g., liposomes, micelles) is more complex and less stable.

### 6.5 Structural designability and environmental responsiveness

The structures of MOFs can be flexibly tailored by adjusting metal ion types, ligand species, and synthesis conditions, enabling intelligent response to the microenvironment (e.g., pH, temperature, enzymes) (Choi and Paul, 2025). For instance, in the acidic microenvironment of bone infection, MOFs can rapidly degrade and release antibiotics; in the neutral environment of normal bone tissue, they remain stable to reduce drug waste (Feng et al., 2024). This responsiveness is significantly superior to PLGA or CS nanoparticles, which lack environmental sensitivity.

## 7 Addressing challenges in MOF application and strategies to enhance osteogenic capabilities

**FIGURE 8:**
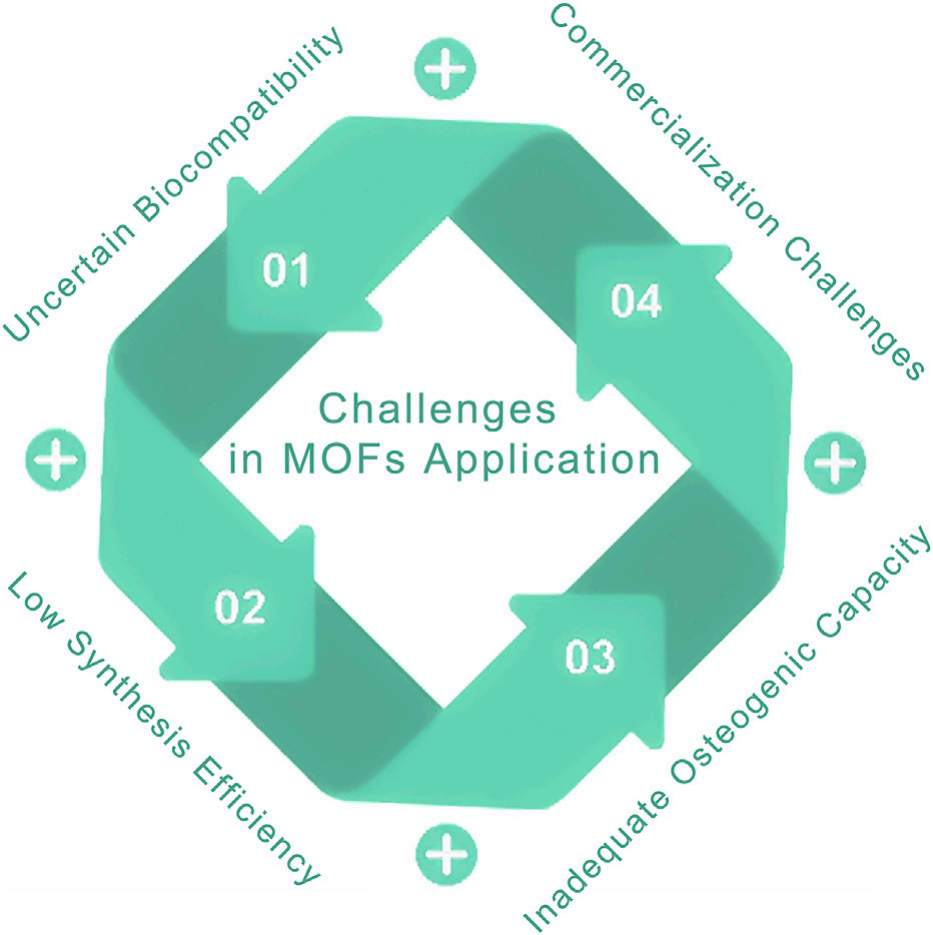
Challenges affecting the application scope of MOFs.

Although MOFs have demonstrated promising osteogenic effects in experimental stages, their clinical application and development face challenges such as uncertain biocompatibility, low synthesis efficiency, inadequate osteogenic capacity to meet clinical demands, and hindered commercialization due to the complexity of the human microenvironment (Figure 8). Consequently, researchers have extensively explored strategies to enhance the osteogenic performance of MOFs in recent years. This section will discuss the measures to address these challenges in detail.

### 7.1 Strategies to address uncertain biocompatibility of MOFs

Biocompatibility is one of the most critical considerations in biomaterial design, indicating a material’s ability to carry out its function without triggering toxic or harmful reactions in biological systems while inducing an appropriate host response under specific conditions (Lu et al., 2023). Today, the concept of biocompatibility encompasses not only bio-inertness but also biofunctionality and bio-stability (Huzum et al., 2021), such as hemocompatibility, histocompatibility, and immune responses. However, a primary concern for MOF-based nanomaterials is their uncertain biocompatibility. Due to the diverse physicochemical properties of MOFs and their complex interactions with cells, efficacy does not equate to safety. Many MOFs still face biocompatibility-related challenges. For instance, the surface charge of MOF nanoparticles can attract plasma proteins, leading to unusual blood clotting and the breakdown of red blood cells (Zheng et al., 2024). Toxicity can also be caused by the excessive local release of metal ions or drugs from MOFs.

Jiang et al. synthesized micro/nano-scale bio-functional entities (m/n-bio-MOF-1) using a modified method and systematically evaluated them across multiple dimensions, covering cell multiplication, oxidative stress, apoptosis, and animal trials (Jiang et al., 2022). Reducing the concentration of n-bio-MOF-1 resulted in smaller particles, which enhanced cell adhesion, improved morphology, lowered *in vitro* cytotoxicity, decreased ROS induction, and increased stability. Beyond modifying the MOF framework itself, surface functionalization can also enhance biocompatibility. For instance, PEGylation (polyethylene glycol modification) of MOFs improves intracellular stability and enables delayed drug release with reduced cytotoxicity (Chen X. et al., 2021; Li et al., 2023b).

Therefore, two critical aspects warrant consideration when improving the biocompatibility of MOFs. First and foremost, it is critical to assess the metabolic profiles of MOF particles *in vivo* to clarify whether their metabolic byproducts pose health hazards and to guarantee their safe and efficient excretion (Singh et al., 2021). A second key consideration centers on the hemocompatibility of MOF particles, which may trigger blood coagulation, hemolysis, or abnormal platelet activation upon administration. The hemocompatibility of MOF particles is primarily shaped by parameters including surface charge, size, and morphology, which are amenable to optimization via targeted modifications.

### 7.2 Strategies to address low synthesis efficiency of MOFs

Apart from ensuring biocompatibility, the production of MOFs with high purity is a major challenge for their market and medical application. In biomedical applications, MOF purity is paramount. However, conventional synthesis methods often involve toxic solvents and impurities, necessitating the development of green synthesis approaches. Solvothermal methods and post-synthetic modifications frequently suffer from inconsistent reaction conditions (temperature, pressure, precursor ratios), leading to insufficient product purity. In catalytic applications, improper design of MOF pore structures—such as excessively narrow pores or uneven metal loading—can trap intermediate products, triggering side reactions and complicating the isolation of pure MOF materials. In this scenario, laser-induced synthesis has become a revolutionary technology (Guo et al., 2023). This high-efficiency pyrolysis technique enables the production of purer MOFs compared to traditional hydrothermal/solvothermal methods. Major benefits are quick processing, accuracy, minimal waste, high efficiency, selectivity, and programmability, which give MOF derivatives new functionalities. Laser-synthesized MOFs show improved crystallinity and defect management, positioning them as promising options for cutting-edge biomedical and catalytic uses.

### 7.3 Strategies to address inadequate osteogenic capacity of MOFs for clinical needs

The precise modulation of MOF parameters is crucial for attaining desired therapeutic outcomes in osteogenic treatments (Manzari et al., 2021). Critical factors, including MOF concentration, pore size, drug loading capacity, and active site configuration, necessitate meticulous optimization. Furthermore, improving the targeting specificity of MOFs for various osteogenic-disrupting pathologies is vital to ensure accurate recognition of diseased tissues. MOFs that incorporate multiple osteogenic mechanisms typically demonstrate enhanced laboratory performance and exhibit greater potential for clinical translation. In the following discussion, we concentrate on strategies for adjusting pore size.

Osteogenic MOFs are taken up by osteoblasts in a manner significantly influenced by the size effect. Cells tend to internalize smaller-sized nanoparticles, suggesting that rational adjustment of MOF dimensions can enhance their uptake by bone-related cells (Xue Y. et al., 2021). Additionally, miniaturization increases interactions between MOFs and cellular components, thereby boosting osteogenic capabilities (Batool et al., 2023). Ahemed et al. successfully synthesized Zn-MOF/bioactive glass (BG) nanoparticles using a rapid alkali-modified sol-gel method (Ahmed et al., 2024). In comparison to standalone BG, the BG/Zn MOF composite demonstrated superior antimicrobial activity and prolonged drug release. Nano-ZIF-8 membranes further enhanced ALP activity, extracellular matrix mineralization, and osteogenic gene expression. Compared to larger Zn-MOFs, the nano-sized Zn-MOFs exhibited higher specific surface area and improved dispersibility. Moreover, they facilitated hydroxyapatite formation, contributing to effective bone regeneration. Nonetheless, the efficient cellular absorption of nano-sized MOFs suggests they might infiltrate cell membranes and gather in healthy cells, which could be hazardous to human health.

By carefully designing the pore sizes of MOFs, their effectiveness as carriers for drug molecules, such as osteogenic inducers and antibiotics, can be improved to aid in bone regeneration. For successful drug incorporation, the pore dimensions of MOFs should match the size of drug molecules, which can differ. Deng and colleagues created an innovative multifunctional photosensitive yolk-shell nanoparticle that responds to stimuli by selectively etching a biodegradable crystalline ZIF-8 shell around a star-shaped gold nanoparticle photothermal yolk using tannic acid (Deng et al., 2019). The findings indicated that the ZIF-8 shell became significantly thinner, creating additional space between the Au nanoflowers and the MOF framework. Compared to core-shell ZIF-8@Au structures, this yolk-shell configuration exhibited substantially enhanced drug-loading capacity.

### 7.4 Challenges in MOF commercialization

MOFs have not yet been successfully commercialized within biomedical applications. One of the core obstacles to the commercialization of MOFs is the high production cost and significant difficulty in scaling up, which mainly stem from the cost of raw materials, synthesis efficiency, and the complexity of downstream processing (Barsoum et al., 2025). In the field of biomedicine (such as bone implantation), commercialization needs to address issues like metal ion toxicity, controllability of degradation, and immune responses (Wright et al., 2025). Moreover, it is necessary to optimize performance for specific scenarios; for example, bone implants require a balance between mechanical strength and osteoconductivity (Chen Z. et al., 2021). Finally, there exist problems in the MOF field such as a disconnect between research and industrialization, as well as insufficient interdisciplinary collaboration,all of which have impeded the widespread adoption of MOFs in the biomedical sector (Liu Y. et al., 2024). Nonetheless, with ongoing advancements in MOF research, it is anticipated that their biocompatibility will continue to improve, and biosafety will be further enhanced, thereby establishing a foundation for future biomedical applications. Although MOFs possess significant potential for medical applications, existing limitations must be addressed. Progress in research and technology is expected to help overcome these challenges, ultimately unlocking the precise therapeutic capabilities of MOFs in the field of nanomedicine. Recently, 3D printing technology has been applied to bone implantation. By constructing porous structures with a porosity of 50%–80%, it can match the mechanical properties of cancellous bone (with a compressive strength of 10–30 MPa) while promoting cell infiltration. In addition, composite biomaterials are used, which are compounded with natural polymers (such as collagen and chitosan) or degradable polymers (such as PLGA and PCL). This not only reduces immunogenicity but also matches the bone regeneration cycle (usually 8–12 weeks) (Li M. et al., 2022; Pourmadadi et al., 2023).

## 8 Conclusions and perspectives

Over recent years, MOFs and MOF-based composite materials have exerted notable influences within the biomedical domain. MOFs, composed of metal ions and organic ligands, are characterized by their high specific surface area, porosity, atomic utilization efficiency, drug-loading capacity, and excellent biomimetic structures and mechanical properties. Leveraging these advantages, MOFs can be employed to develop highly sensitive biosensors. Many diseases causing osteogenesis disorders are associated with changes in specific biomarkers, such as rheumatoid factor, blood glucose, ALP, and certain cytokines. Real-time monitoring of these biomarkers using biosensors could serve an important purpose in disease treatment and prognosis management (Flynn et al., 2023). In one instance, Xu and their team designed a glucose sensor that does not rely on enzymes by directly growing conductive Ni/Co-based carbon cloth [Ni/Co(HHTP) MOF/CC], achieving remarkable results in glucose detection within real serum samples (Xu et al., 2021). Additionally, Xu et al. synthesized a novel europium-centered MOF [(CH_3_)_2_NH_2_][Eu(cdip)(H_2_O)] for uric acid detection, offering substantial benefits for gout patient management (Xu et al., 2023). These advancements suggest that designing functional MOF-based biosensors for detecting OP, rheumatoid arthritis (RA), and bone infections is feasible, enabling localized monitoring of disease progression.

Excellent targeting ability and controlled release are crucial functions of MOFs as drug delivery systems, particularly in therapies aimed at promoting bone repair. Therefore, continuously enhancing the specificity of MOFs in recognizing diseased tissues can improve therapeutic efficacy while minimizing impacts on healthy tissues. The research by Deng et al. involved delivering celastrol (CEL) to arthritic joints to specifically induce apoptosis in osteoclasts and macrophages (Deng et al., 2021). They employed enzyme-responsive nanoparticles, known as PRNPs, which consist of RGD-modified nanoparticles (RNPs) covered with PEG chains that can be cleaved. CEL-PRNPs targeted both osteoclasts and inflammatory macrophages in RA patients by interacting with RGD-αvβ3 integrin following MMP-9 cleavage of PEG, resulting in increased apoptosis of these cells. Additionally, utilizing the sensitivity of MOFs to factors like pH and ROS, the system enables multi-targeted therapy for RA, resulting in improved therapeutic outcomes, inflammation alleviation, and bone erosion repair.

However, the biomedical applications of MOFs still have limitations. First, the controllability of degradation is insufficient, and long-term safety is questionable. They rely on an acidic microenvironment for degradation but degrade too slowly in a neutral physiological environment, which may lead to foreign body retention. Accumulation of MOF degradation products (such as Zn^2+^ and Co^2+^) may trigger chronic inflammation. Zirconium-based MOFs have been reported to have high cytocompatibility, but the renal excretion pathway of Zr^4+^ is unclear, and large-dose implantation may cause metal accumulation. The nickel ion (Ni^2+^) release threshold (e.g., > 50 μg/mL) of β-CDs/Ni-based MOF scaffolds may induce allergic reactions. Second, the long-term ion release profiles lack dynamic data in physiological environments. Although short-term release is controllable, long-term behavior remains unclear. Most studies only report release profiles over 7–28 days, while the bone regeneration cycle lasts 3–6 months. There is a lack of data from large animal models regarding whether the release attenuates or bursts over time. Furthermore, there are risks in clinical translation. The mass production technology is immature; for example, electrospinning and 3D printing rely on laboratory-scale processes, and during scale-up production, problems such as non-uniform MOF particle sizes and fluctuations in scaffold porosity arise. The solvothermal method (e.g., UiO-66 synthesis requiring DMF) has residues of toxic solvents and high energy consumption, failing to meet GMP standards. Meanwhile, there is a lack of production regulatory standards. As a new type of material, MOFs lack FDA/EMA guidelines regarding the toxicity thresholds of their degradation products (e.g., daily tolerable intake of Zr^4+^) and immunogenicity (e.g., whether MOF crystals trigger foreign body giant cell reactions). Gaps in preclinical research have left key mechanisms and long-term effects unclear. Existing studies focus on short-term immunomodulation, but there is a lack of data beyond 12 weeks regarding the sustained impact of MOF degradation products on macrophage phenotypes and their recruitment effects on T cells/neutrophils. The early mechanical strength of MOF composite scaffolds (with a compressive modulus of 10–50 MPa) can meet the needs of cancellous bone, but the modulus decreases by more than 50% after 6 weeks as MOFs degrade, which may lead to implant collapse. Currently, there is a lack of research on the three-way dynamic matching of “degradation-mechanics-osteogenesis.” There is no epidemiological evidence regarding whether long-term release of Ag^+^ from antibacterial MOFs (>6 months) induces drug-resistant bacteria, or whether chronic inflammation caused by zirconium-based MOFs increases the risk of osteosarcoma.

What does the future hold for MOFs, and how can we unlock the full potential and power of this emerging chemical field? Digital Reticular Chemistry: With digital computing technology continuing to progress, computational chemistry has emerged as a critical element in modern chemistry. Within the realm of reticular chemistry, the significance of computational chemistry resides in its capacity to ascertain molecular-scale structures and predict new architectures boasting improved performance across various application contexts. Incorporating artificial intelligence (AI) into computational chemistry holds the potential not only to revolutionize the domain of reticular chemistry but also to ignite a transformative wave across the broader sphere of chemical research. However, it is important to note that AI can only operate within the chemical space covered by its training data and cannot make reliable inferences about content beyond that space. Therefore, researchers still need to rely on their professional knowledge and experience to make critical decisions during the exploration process, determining when to introduce significant innovations (Gagliardi and Yaghi, 2023).

Imagine if we could leverage large language models (LLMs) such as GPT-4 to reliably mine information—for instance, compiling the reaction conditions required for the synthesis and crystallization of MOFs, or using machine learning algorithms to predict new MOFs and link their structures to specific properties and biomedical applications. This would undoubtedly open up more possibilities for future disease treatments. Thanks to these technological advancements, information can be shared, collaborations can be advanced, and knowledge can be co-created, enabling researchers to explore and analyze MOFs regardless of their geographical location.

This review provides a comprehensive summary of recent advancements in the development of MOFs for enhancing bone regeneration, emphasizing their varied applications within this domain. MOF-based nanomaterials demonstrate remarkable potential in facilitating bone healing by promoting osteogenesis, modulating inflammatory responses, exerting antioxidant effects, offering antimicrobial properties, and stimulating angiogenesis. Moreover, specific MOFs demonstrate significant therapeutic potential by synergistically incorporating multiple factors that promote osteogenesis. Researchers have engineered a variety of functionalized MOF-based nanomaterials for the treatment of bone-related diseases. Nonetheless, several challenges persist, including the necessity for process optimization, variability in biocompatibility, and the absence of standardized protocols and certifications for clinical application. Continued research and advancements in production technologies are crucial to enable the clinical utilization of MOFs in therapies designed to enhance bone regeneration.
